# Deciphering the microbial community structures and functions of wastewater treatment at high-altitude area

**DOI:** 10.3389/fbioe.2023.1107633

**Published:** 2023-02-27

**Authors:** Yuliang Zhu, Yucan Liu, Huanhuan Chang, Hao Yang, Wei Zhang, Yanxiang Zhang, Hongwei Sun

**Affiliations:** ^1^ School of Environmental and Material Engineering, Yantai University, Yantai, China; ^2^ School of Civil Engineering, Yantai University, Yantai, Shandong, China; ^3^ School of Environmental and Municipal Engineering, Lanzhou Jiaotong University, Lanzhou, China

**Keywords:** Illumina High-throughput sequencing, high-altitude WWTPs, microbial communities, core bacteria, correlation network analysis

## Abstract

**Introduction:** The proper operation of wastewater treatment plants is a key factor in maintaining a stable river and lake environment. Low purification efficiency in winter is a common problem in high-altitude wastewater treatment plants (WWTPs), and analysis of the microbial community involved in the sewage treatment process at high-altitude can provide valuable references for improving this problem.

**Methods:** In this study, the bacterial communities of high- and low-altitude WWTPs were investigated using Illumina high-throughput sequencing (HTS). The interaction between microbial community and environmental variables were explored by co-occurrence correlation network.

**Results:** At genus level, *Thauera* (5.2%), *unclassified_Rhodocyclaceae* (3.0%), *Dokdonella* (2.5%), and *Ferribacterium* (2.5%) were the dominant genera in high-altitude group. The abundance of nitrogen and phosphorus removal bacteria were higher in high-altitude group (10.2% and 1.3%, respectively) than in low-altitude group (5.4% and 0.6%, respectively). Redundancy analysis (RDA) and co-occurrence network analysis showed that altitude, ultraviolet index (UVI), pH, dissolved oxygen (DO) and total nitrogen (TN) were the dominated environmental factors (*p* < 0.05) affecting microbial community assembly, and these five variables explained 21.4%, 20.3%, 16.9%, 11.5%, and 8.2% of the bacterial assembly of AS communities.

**Discussion:** The community diversity of high-altitude group was lower than that of low-altitude group, and WWTPs of high-altitude aeras had a unique microbial community structure. Low temperature and strong UVI are pivotal factors contributing to the reduced diversity of activated sludge microbial communities at high-altitudes.

## 1 Introduction

At present, activated sludge (AS) is the most widely used wastewater biological treatment technology in wastewater treatment plants (WWTPs). AS is a very complex biological system with great diversity (Zhang et al., 2012). Microorganisms in AS play an important role in the stable operation of WWTPs (Ju and Zhang, 2015; Li and Pagilla, 2017; Zhang et al., 2020). The activity and interaction of microorganisms are considered key factors in biological treatment technology (Zhang et al., 2017), and the removal efficiency of pollutants is usually positively correlated with microbial diversity (Zhang Y. et al., 2016; Fang et al., 2018). The microbial community structure is affected by many factors, such as spatial position (Saunders et al., 2016; Fang et al., 2018), climate conditions (Fang et al., 2018; Petrovski et al., 2020), characteristics of sewage (Chen et al., 2020; Shen et al., 2020), process facilities (Gao et al., 2016), and operating parameters [temperature (Petrovski et al., 2020) and dissolved oxygen (DO) (Wells et al., 2011)]. Currently, High-throughput sequencing (HTS) technology is the most effective method for improving the breadth and depth of the microbial community. Currently, HTS is widely used in the analysis of community structure in soil (Shi et al., 2021), wastewater treatment (Wang et al., 2012), and other natural environments (Wu et al., 2017). Saunders et al. (2016) analyzed the microbial communities of 13 WWTPs in Denmark using Illumina HTS and found that the community structure of WWTPs varied across regions. However, most studies have focused on the community structure of WWTPs at low-altitude areas, and few studies have been conducted on the microbial community characteristics at high-altitude WWTPs. Considering the harsh climatic conditions at high-altitude areas, it is assumed that the AS system may have a unique microbial community composition.

The ecosystem in high-altitude cold areas is fragile, and the self-purification ability of river and lake water bodies is poor (Qiu 2014). The normal operation of WWTPs is crucial to the protection of the environment of rivers and lakes (Jin et al., 2014; Zhang Q. H. et al., 2016). The local temperature and DO in water decrease with an increase in altitude. However, the intensity of the ultraviolet index (UVI) increases (Fang et al., 2018). These parameters are critical to the diversity and abundance of microbial composition (Kang et al., 2020). Particularly, higher UVI can damage the DNA or RNA molecular structure of microorganisms, making them less active or even dead (Fang et al., 2018). It has been reported that the proportion and function of denitrifying bacteria decreased significantly with the increasing altitude of WWTPs (Niu et al., 2016). Fang et al. (2018) found that microbial diversity was negatively correlated with altitude and positively correlated with water temperature. Kang et al. (2020) analyzed microbial community differences at different altitudes and showed that temperature and DO were the main factors driving community composition at high altitudes. However, these studies mainly focused on the effect of altitude and temperature on the microbial community structure, and the effect of UVI has been neglected.

The reduced efficacy of WWTPs at high-altitude areas is largely due to changes in the microbial community of AS. However, the core species and their contribution to AS remain to be explored. The co-occurrence correlation network will deepen the understanding of microbial communities beyond simple diversity and composition. In this work, the diversity and structure of the microbial community of AS in WWTPs were characterized using HTS, and the main environmental factors affecting the composition of the AS community and the core species were explored using co-occurrence correlation network analysis.

In this study, AS samples were collected from six WWTPs (three high-altitude and three low-altitude WWTPs) in China. The microbial community diversity and composition were analyzed by Illumina HTS. This work intended to i) evaluate the changes in microbiota diversity and composition in high- and low-altitude regions, ii) characterize the distribution of functional and core bacteria in different WWTPs, and iii) reveal the main drivers affecting microbial community assembly. This study may provide a valuable reference for improving the treatment efficiency of WWTPs in high-altitude areas.

## 2 Materials and methods

### 2.1 Description of WWTPs and sample collection

For this study, six municipal WWTPs located in two different geographical locations in China: Shandong province (Qingdao and Yantai, 10–22 m above sea level) and Gansu province (Lanzhou, 1,520–1,708 m) were selected. There was approximately a 1,500 m elevational difference between the Shandong and Gansu provinces, and they were regarded as the typical provinces of eastern and western China, respectively. The general basic information and wastewater characteristics of the six WWTPs are shown in Table 1. Among them, three WWTPs were located in Yanerwan (YEW), Heping (HP), and Yanchangpu (YCP) at high altitudes in Lanzhou; one was located in Xin’anhe (XAH) in Yantai; and two were located in Jimo (JM) and Chengyang (CY) at low altitudes. Detailed information about the WWTPs and the samples are provided in Table 1. Samples were taken for high-altitude WWTPs in November 2019 and for low-altitude WWTPs in December 2019 and January 2020 to minimize the impact of wastewater temperature on resistance genes. The average wastewater temperatures in high-altitude (15.7°C ± 1.1°C) and low-altitude (15.0°C ± 1.2°C) regions were similar, three parallel samples were collected from AS systems in high-altitude WWTPs, whereas two parallel samples were collected from low-altitude WWTPs. Approximately 5 ml of water sample was collected and then mixed with 5 ml of anhydrous ethanol in a 10 ml sterile polyethylene centrifuge tube. These fixed samples were stored in an incubator with an ice bag, transported to the laboratory, and stored in a refrigerator at −80°C for subsequent DNA extraction. DO, temperature, and pH were measured immediately after collection using the pH/oxi 340 analyzer (WTW Company, Germany). Altitude was obtained from Google Maps (http://www.gugeditu.net/). UVI was determined from the Weather Online platform (https://www.woeurope.eu/) for 15 days before and after sampling.

**TABLE 1:** Basic overview of six WWTPs surveyed in China.

Group	WWTP code	Sample code	Longitude and latitude	Flow (10^4^ m^3^/d)	SRT (days)	HRT (hours)	Percentage of industrial wastewater	Treatment process	Altitude (m)	UVI	Bioreactor		
											T (°C)	pH	DO (mg/L)
High-altitude	YEW	YEW-1	(103°54′E36°02′N)	26	19.7	12.8	20	A/A/O	1520	3.26	16.8	8.12	3.83
		YEW-1											
		YEW-1											
	YCB	YCB-1	(103°85′E36°07′N)	4	15.3	11.5	0	CASS	1699	3.30	15	7.62	2.23
		YCB-1											
		YCB-1											
	HP	HP-1	(104°11′E35°84′N)	11	18.5	12.3	2	OD	1708	3.35	14.7	8.12	2.23
		HP-1											
		HP-1											
Low-altitude	XAH	XAH-1	(120°52′E37°60′N)	12	12.3	7.9	20	A/A/O	57	1.79	16	6.81	4.93
		XAH-1											
	CY	CY-1	(120°40′E36°31′N)	5.5	10.9	9.3	60	CASS	10	1.75	13.8	7.2	6.5
		CY-1											
	JM	JM-1	(120°45′E36°39′N)	17	13.3	8.5	30	OD	22	1.76	15	7.14	1.8
		JM-1											

SRT, sludge retention time; HRT, hydraulic retention time; UVI, ultraviolet index; MLVSS, mixed liquor volatile suspended solid; A/A/O, anaerobic/anoxic/aerobic; CASS, cyclic activated sludge system; OD, oxidation ditch.

### 2.2 DNA extraction and PCR amplification

Genomic DNA was extracted by FastDNA^®^ SPIN Extraction Kit (MP, Biomedicals, United States) following the manufacturer’s instructions. DNA quality was assessed by 1% agarose gel electrophoresis, and the concentration and purity of DNA were determined using the NanoDrop spectrophotometer (nd-1000, NanoDrop, United States). 16S rRNA gene universal primers set 338F (5′-ACT​CCT​ACG​GGA​GCA​G-3′) and 806R (5′-GGACTACHVGGGTWTCTAAT-3′) were used to amplify the V3–V4 variable region of DNA. The PCR amplification system was constructed, including 95°C pre-denatured for 5 min, denatured at 95°C for 30 s, annealed at 55°C for 30 s, extended at 72°C for 45 s, cycled 30 times, and finally elongated at 75°C for 10 min. Each sample was amplified by three-parallel amplification. After amplification, the PCR product was detected on 2% (w/v) agarose gel, and the size of each amplicon was not less than 550 bp. PCR amplicons were extracted from the 2% agarose gel, purified by the AxyPrep DNA gel Extraction Kit (Axygen Biosciences, United States), and quantified by the QuantiFluor™- ST (Promega, United States). Equal molar quantities of purified amplicon were collected, then paired-end sequences were performed using the MiSeq PE300 platform (Illumina, United States) (2 × 250). The raw data were deposited in the NCBI Sequence Read Archive (SRA) with the accession number SUB10611130.

### 2.3 Analysis of microbial community diversity

The USEARCH 7.1 (Caporaso et al., 2012) was used for quality control and filtering of the raw sequence. The main steps were discarding short sequences less than 50 bp, removing repetitive sequences, and discarding chimeric sequences. The optimized sequences were clustered into an operational taxonomic unit (OTU) based on 97% sequence similarity using UPARSE 7.1 (Edgar 2013). The minimum number of reads at six samples was selected to statistically analyze all samples at the same sequencing depth. Then, the effective sequences were compared with the SILVA database (http://www.arb-silva.de). According to OTU information, the α-diversity index, including the community diversity index (Shannon, Simpson, and Chao1), community evenness index (Pielou) (Sun et al., 2021a), and sequencing depth index (Good’s coverage), were calculated using MOTHUR (http://www.mothur.org/wiki). The α-diversity of the microbial communities was expressed using the Chao1, Pielou, Shannon, and Simpson diversity indices. The Chao1 index was used to evaluate community richness, and both the Simpson and Shannon indices could be used to evaluate community diversity. Pielou was performed to estimate the evenness of the community (Sun et al., 2021a). A larger α-diversity value represents higher richness and diversity of the bacterial community. Principal component analysis (PCA), the most common method of ordination analysis, can be used to demonstrate differences in species abundance composition with relatively small numbers of species at a given taxonomic level (Ramette, 2007). Therefore, PCA and hierarchical cluster analysis were performed to visualize the relationships of the microbial community based on Bray–Curtis distance using QIIme 1.7.0. The redundancy analysis (RDA) was performed using Canoco 4.5.

### 2.4 Statistical analysis

All data on reaction rates, providing averages and standard deviations, were calculated using Microsoft Excel software (v. 2019, Microsoft, United States). The Microbial Database for Activated Sludge (MiDAS) was used to screen potential functional genera (Nierychlo et al., 2020). Independent *t*-tests were used to compare the mean differences in microbial diversity and functional genera between the two groups, and Spearman’s correlation analysis was applied to explore potential correlations between bacterial and environmental factors. All statistical analyses were performed on SPSS software (version 20.0), and the results were considered to exhibit significant differences when *p* < 0.05. Gephi software was used to visualize the co-occurrence correlation network based on Spearman’s analysis.

## 3 Results and discussion

### 3.1 The treatment profile and performance of WWTPs

The daily wastewater treatment capacity varied from 2.6 × 10^5^ m^3^/d to 5.0 × 10^5^ m^3^/d in the six WWTPs. The wastewater comprised approximately 70%–80% domestic wastewater and nearly 20%–40% industrial wastewater in WWTPs, except for YCB, which comprised 100% domestic wastewater.

The overall treatment performance characteristics of the WWTPs are summarized in Supplementary Table S1. Similar average removal efficiencies of COD and TP in the high-altitude and low-altitude groups were observed, and the removal efficiencies of NH_4_^+^-N and TN in the low-altitude group were 2.7%–9.3% higher than those in the high-altitude group (Supplementary Table S1). This result was consistent with the conclusion obtained by Fang et al. (2018), who found that the nitrogen removal performance in plateau WWTPs was relatively lower. A reasonable and common explanation for this was the low DO concentration and low regional temperature in plateau regions. DO and temperature are the key factors influencing the biological treatment of wastewater (Niu et al., 2016; Wu et al., 2019). The intrinsic mechanism of microbial community structure change driven by DO is that the oxygen affinity varies for different microorganisms, and low DO concentration can inhibit the activity of AOB (Chen et al., 2017), thus reducing the effectiveness of nitrogen removal. The diversity and activity of the microbial community will be decreased when the AS system suffers from long-term low-temperature impact, leading to performance deterioration of the process (Chen et al., 2017; Li et al., 2021). In addition, Wu et al. (2019) analyzed approximately 1,200 AS samples from 269 WWTPs in 23 countries on six continents and showed that temperature was the main environmental variable shaping AS bacterial composition on a global scale, with a negative correlation with species richness. The pollutant removal in biological WWTPs essentially depended on the diversity and community of the functional microbial population of AS system.

### 3.2 Multiple environmental factors co-affected the microbial community composition

Among all AS samples, the number of effective sequences ranged from 42,466 to 65,148, and OUT varied from 2,987 to 4,461 (Supplementary Table S2). Good’s coverages (0.983–0.993) of all samples were greater than 0.980, indicating that the sequencing depths were enough for further analysis. All sample sequences tended to be saturated in the rarefaction curve (Supplementary Figure S1), indicating that the sequencing depths were sufficient.

**FIGURE 1:**
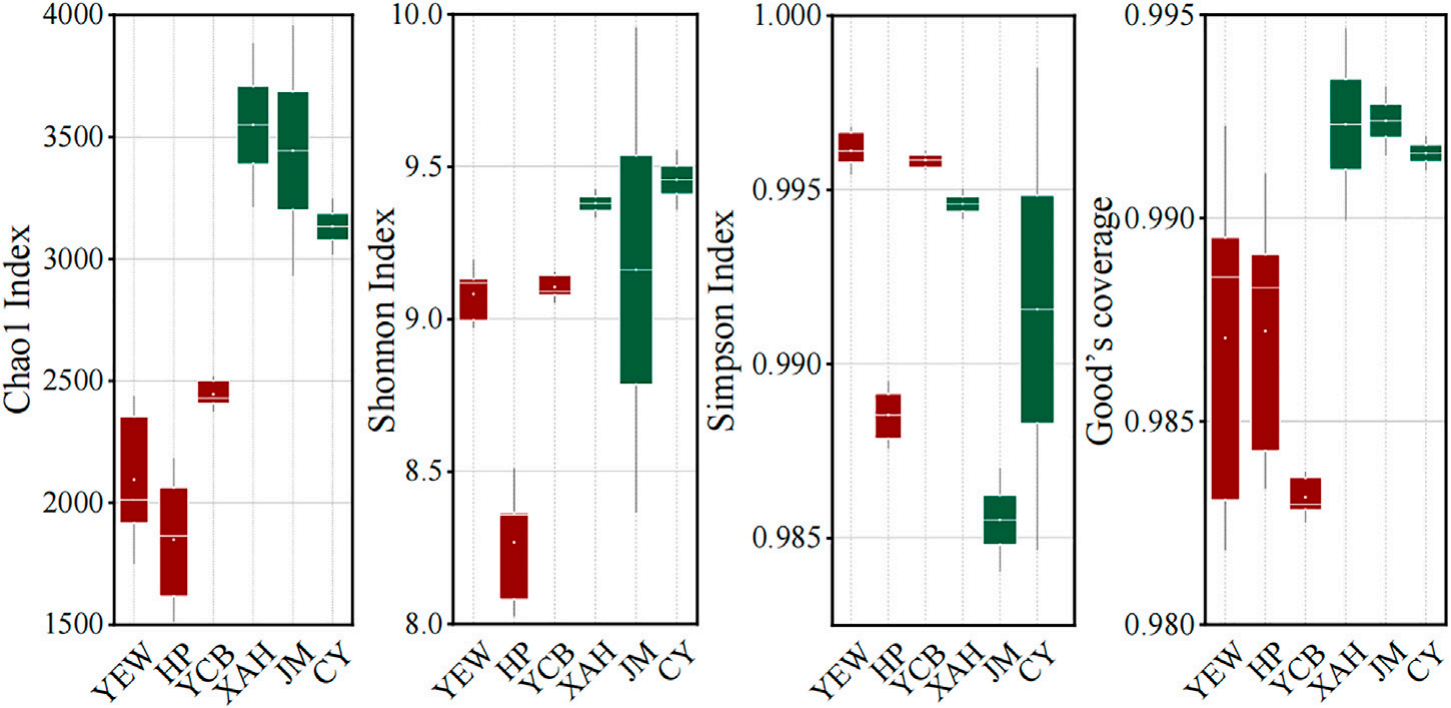
Microbial α-diversity of the activated sludge system taken from six real municipal wastewater treatment plants (WWTPs). The red and green boxes represent high- and low-altitude samples, respectively.

The *α*-diversities of the microbial communities were expressed using the Chao1 estimator, Shannon index, and Simpson index, which reflect the richness, evenness, and diversity of the microbial community (Shannon, 1948; Simpson, 1949; Chao, 1984). A larger *α*-diversity value represents higher richness and diversity of bacterial community (Sun et al., 2020). The high-altitude group had significantly lower effective sequences number (*p* < 0.05) and OTU number (*p* < 0.05) than the low-altitude group. Both Chao1 and Shannon indexes in the low-altitude group were significantly higher (*p* < 0.05) than those in the high-altitude group, indicating that low-altitude WWTPs displayed higher richness and evenness of the microbial community. However, the Simpson index was significantly higher (*p* < 0.05) in the high-altitude group (Figure 1), demonstrating that high-altitude WWTPs harbored more abundant diversities of the microbial population. These results clearly demonstrated that the altitude had an important effect on the α-diversity of the microbial communities of AS.

**FIGURE 2:**
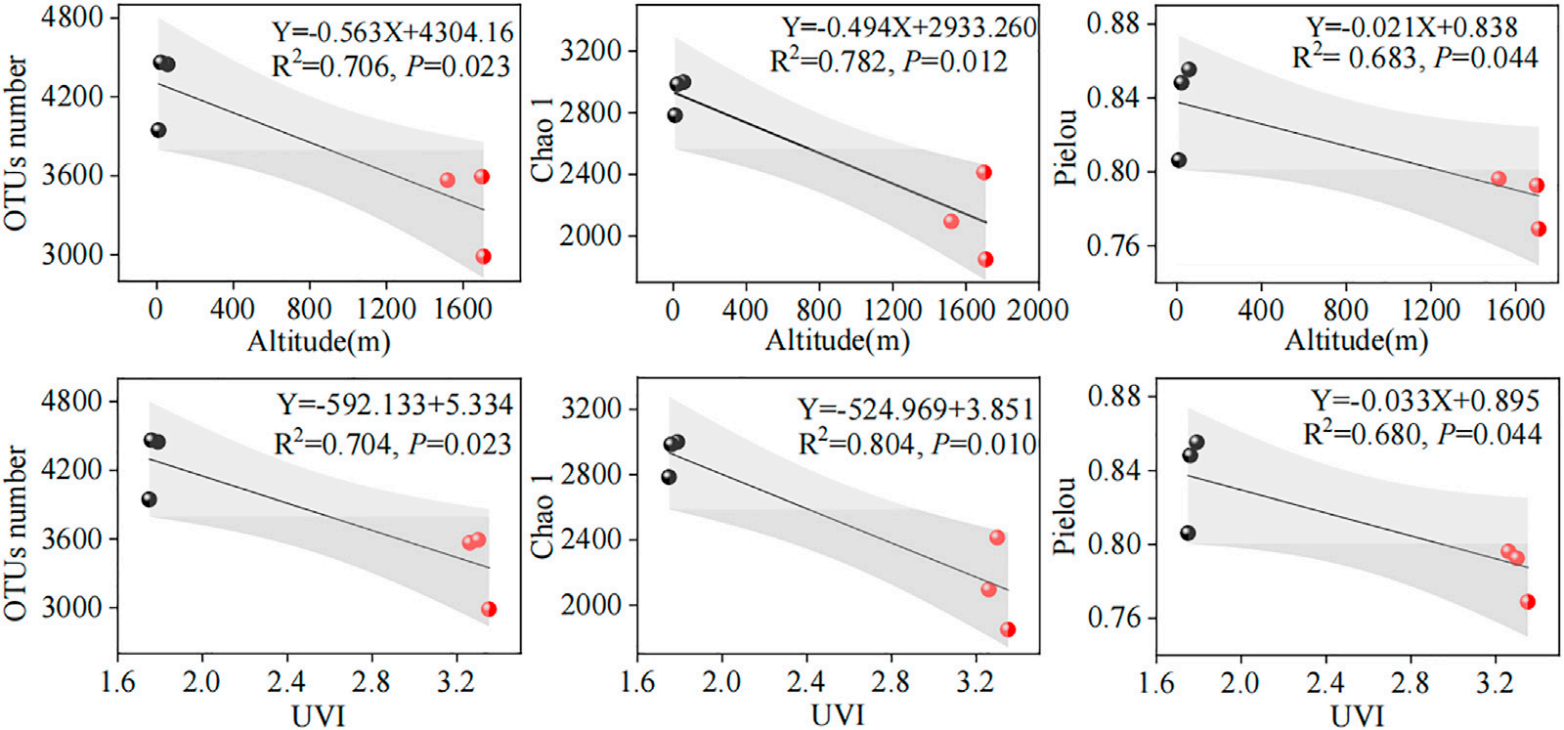
Linear correlations between microbial diversity and altitude and UVI. The red and black dots represent high- and low-altitude samples, respectively. The gray part represents the 95% confidence interval of the linear model.

The relationship between microbial diversity and environmental factors (altitude and UVI) could be revealed using linear regression analysis (Figure 2). In this study, parallel samples from different WWTPs were averaged and subjected to linear regression analysis. We acknowledged that the linear correlations between microbial diversity and altitude and UVI were slightly weak, with an *R* ^2^ range of 0.683–0.804. A reasonable explanation for this is that the microbial diversity was affected by the synergistic effect of various elements, such as environmental factors, operating parameters, and influent characteristics, supported by the PCA result in Section 3.5. The results showed that OTU number, Chao1, and Pielou indexes were significantly and negatively correlated with altitude (*R* ^2^ = 0.706, *p* < 0.05; *R* ^2^ = 0.782, *p* < 0.05; and *R* ^2^ = 0.683, *p* < 0.05, respectively) (Figure 2) and UVI (*R* ^2^ = 0.704, *p* < 0.05; *R* ^2^ = 0.804, *p* < 0.05; and *R* ^2^ = 0.680, *p* < 0.05, respectively) (Figure 2). This observation was consistent with those obtained by Fang et al. (2018), who investigated microbial communities of plateau WWTPs and found that microbial diversities and richness were negatively associated with the altitude. However, Wilhelm et al. (2015) investigated the altitudinal patterns of diversity of metabolically active microorganisms in stream biofilms. Their study showed no significant linear correlation between microbial diversity and geographical altitude. This different observation can mainly be explained by various factors, including the treatment process, wastewater quality, microbial population, and operating parameters.

### 3.3 Microbial community structure and the core taxa of AS system at different altitudes

**FIGURE 3:**
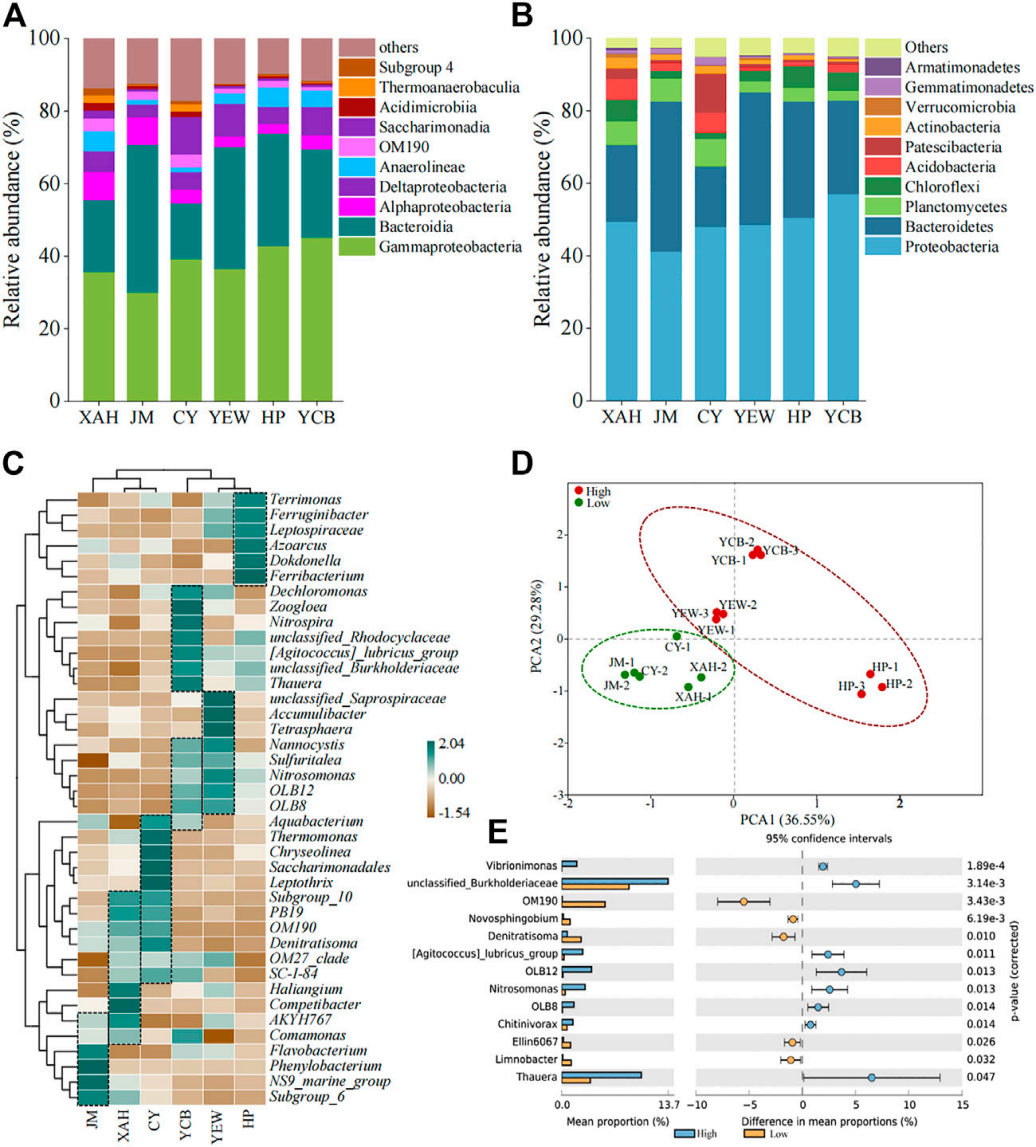
Microbial community composition at the **(A)** phylum level, **(B)** class level, and **(C)** genus level. **(D)** Genera with a significant difference among two groups. **(E)** PCA.

Classification of microorganisms was performed at the phylum and class level. The top 10 bacteria of all samples are shown in Figures 3A, B; Supplementary Tables S3, S4. The microbial community of all AS samples had a high diversity at the phylum level, reaching more than 48 phyla. The dominant phyla were *Proteobacteria* (41.2%–57.0%), *Bacteroidetes* (16.8%–41.4%), *Planctomycetes* (2.9%–7.5%), and *Chloroflexi* (1.8%–6.1%). The other phyla, including *Acidobacteria* (0.8%–5.7%), *Patescibacteria* (0.7%–11.0%), and *Actinobacteria* (0.6%–2.9%), were minor groups. Among all samples, the relative abundance of *Proteobacteria* was the highest, which was consistent with previous studies (Fang et al., 2018). *Planctomycetes* in the low-altitude group (6.3%–7.5%) were significantly higher (*p* < 0.05) than that in the high-altitude group (2.9%–3.9%). So far, all the bacteria with anammox function were from *Planctomycetes* (Sobotka et al., 2017), which was an ecologically friendly microorganism for safe nitrogen removal (van Teeseling et al., 2015). The relative abundances of *Chloroflexi*, *Firmicutes*, *Cyanobacteria*, and *Nitrospirae* in the high-altitude group were higher than that in the low-altitude group. *Firmicutes* had been found to produce spores and could be adapted to extreme environments (Fimlaid and Shen, 2015). Within *Proteobacteria*, *Gammaproteobacteria* dominated (30.1%–45.2%) in all samples, followed by *Deltaproteobacteria* (3.6%–9.1%) and *Alphaproteobacteria* (2.6%–7.7%). According to the latest taxonomy, *Betaproteobacteria* was an order of *Gammaproteobacteria*, namely, *Betaproteobacteriales* (Parks et al., 2018), accounting for 59.2%–84.8% of the total abundance of *Gammaproteobacteria*. Within the *Bacteroidetes* phylum, *Bacteroidia* (15.3%–40.8%) dominated. It is worth mentioning that *Leptospirae* belonging to the *Spirochaete* phylum accounted for 0.2%–1.1% in the high-altitude group while less than 0.1% in the low-altitude group. Overall, the relative abundance of these major phyla was higher in the high-altitude group compared to the low-altitude group, whereas the relative abundance of other minor phyla was generally lower, suggesting that these predominant bacteria played a virtual role in contaminant removal in the high-altitude group.

Furthermore, species at the genus level were further analyzed by clustered heat maps (Figure 3C). The top 40 genera and six samples were clustered based on the Bray–Curtis similarity index. It can be observed that the high-altitude and low-altitude groups were clustered together separately on the columns. PCA also showed a significant difference in the microbial community between high- and low-altitude AS samples. For the relationship within the group, the microbial community structure between XAH and CY was closer than that with JM in the low-altitude group. In comparison, the microbial community structure between YEW and YCB was closer than that with HP in the high-altitude group. Based on biclustering, the genera with higher abundance in each WWTPs were *Unclassified_Saprospiraceae-OLB8* in YEW; *Dechloromonas-Thauera* and *Nannocystis-Aquabacterium* in YCB; *Terrimonas-Ferribacterium* in HP; *Subgroup_10-Comamonas* in XAH; *Aquabacterium-SC-I-84* in CY; and *AKYH767-Subgroup_6* in JM. These genera were the dominant genera in the corresponding sample. *Thauera* (5.2%), *unclassified_Rhodocyclaceae* (3.0%), *Dokdonella* (2.5%), and *Ferribacterium* (2.5%) were the dominant genera in the high-altitude group. *Saccharimonadales* (4.3%), *OM190* (3.2%), *Phaeodactylibacter* (2.9%), *Competibacter* (2.8%), and *Thermomonas* (2.6%) dominated in the low-altitude group. The mean values of the top 50 genera were compared between the two groups using STAMP to distinguish the group differences at the genus level between the low- and high-altitude groups (Figure 3E). Thirteen genera were significantly different (*p* < 0.05), including *Thauera*, *Nitrosomonas*, *Novosphingobium*, and *Denitratisoma*, and eight genera were dominant in the high-altitude group. *Thauera* (3.4%–7.2%) was slightly higher in the high-altitude group than that in the low-altitude group (1.7%–3.0%), which is inconsistent with Fang et al. (2018). *Thauera* was a denitrifying bacterium widely distributed in WWTPs, which could degrade refractory organic matter and had a good survival advantage in a high-altitude and cold environment (Fuchs et al., 2011; Wang and He 2020). *OM190*, belonging to *Planctomycetes*, accounted for 2.3%–3.7% in the low-altitude group, which was much higher than that in the high-altitude group (0%–0.06%).

Although the dominant bacteria in the high-altitude and low-altitude groups differed, most of them had a relatively specific ecological function for the removal of nitrogen and phosphorus and the degradation of organic matter. In addition, most of the functional bacteria were heterotrophic and nitrifying bacteria, such as *Haliangium* (Chen et al., 2016), *Denitratisoma* (Xu et al., 2017), and *Dechloromonas* (Chen et al., 2016). In addition, the AS system was also considered functionally redundant, with different microorganisms performing the same ecological function (Vuono et al., 2015; Zhang et al., 2020). The proportion of industrial wastewater in WWTPs had a certain impact on the microbial structure in AS. The proportion of industrial wastewater influent was higher in CY (60%), and the microbial taxa associated with refractory organic matter degradation, such as *Saccharimonadales* (10.2%), *Thermomonas* (5.3%), and *Leptothrix* (1.9%), were significantly higher (*p* < 0.05) in CY than those in other samples. *Saccharimonadales* (10.2%) were dominant in CY, whereas they only accounted for 0.04%–2.2% in other samples. Studies have found that they existed in the aerobic granular sludge flocculation process and might be able to degrade a variety of polymers (Chen et al., 2021a). However, various microbial compositions were observed in different biological treatment processes (CASS, A/A/O, OD), indicating that the influent characteristics had a greater impact on the microbial community structure than the treatment process, which was consistent with previous studies (Sun et al., 2022).

The core bacterial community was determined according to the occurrence frequency of OTUs in the AS system (Saunders et al., 2016). When OTU appeared in all AS systems, it was defined as a core taxon. Based on a 0.97 sequence similarity threshold, 17,579 OTUs were clustered from six WWTPs, of which 0.8% (135 OTUs) constituted the core taxa, accounting for 14.6%–25.2% (average 20.9%) of sequence abundance (Supplementary Figure S2; Supplementary Table S5). About 55.6% of the core bacteria belonged to *Proteobacteria*, among which 57 OTUs belonged to *Gammaproteobacteria*. The core taxa of *Thauera* had the highest relative abundance, which accounted for 3.1% of the total microbial abundance. The second richest taxon was *Dokdonella*, with a relative abundance of 1.8%. This was supported by Sun et al. (2021b), who investigated the seasonal dynamics of microbial communities in two full-scale WWTPs and found that *Dokdonella* was dominant microbial bacteria. In addition, *Accumulibacter* was the core phosphorus-accumulating organism (PAOs) with a relative abundance of 0.2%. In comparison, Stokholm-Bjerregaard et al. (2017) and Sun et al. (2021b) showed that the core group of PAOs in WWTPs was *Tetrasphaera*. However, Zeng et al. (2018) and Fan et al. (2020) observed that *Accumulibacter* was the most abundant PAOs, which is in good agreement with the present study. The apparent differences in the dominant PAOs in the aforementioned studies were mainly due to differences in wastewater characteristics, process configuration, operating conditions, and geographic location. *Nitrospira* was the core nitrite-oxidizing bacterium (NOB) with a relative abundance of 0.6%. Similarly, Nielsen et al. (2012) observed that Zoogloea was the core denitrifying bacteria for biological nitrogen removal.

### 3.4 Functional microorganisms in AS and prediction of bacterial metabolic function

**FIGURE 4:**
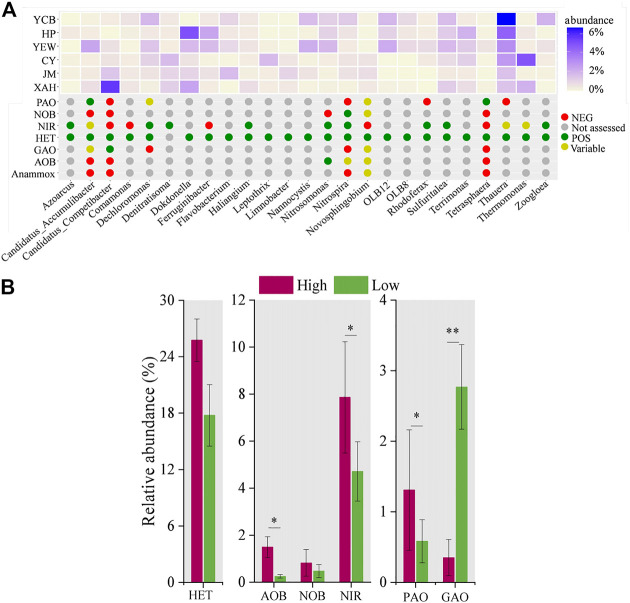
Distribution and relative abundance of functional genera and function prediction among sludge samples. **(A)** Distribution and abundance of functional genera. **(B)** Average abundance of functional genera groups.

MiDAS analysis was used to compare the functional bacteria of high-altitude and low-altitude groups (Van Loosdrecht et al., 2016; Nierychlo et al., 2020). According to the MiDAS database, 25 functional genera among the top 50 dominant genera were screened, and there were significant differences between the two groups (*p* < 0.05) (Figure 4A). The functional genera were further classified into aerobic heterotrophic bacteria (*n* = 24) ammonium-oxidizing bacteria (AOB, *n* = 1), NOB (*n* = 1), nitrite-reducing bacteria (*n* = 9), PAOs (*n* = 2), and glycogen accumulating organisms (GAOs, *n* = 1). Among them, six genera (*Dechloromonas*, *Nitrospira*, *Nitrosomonas*, *Thauera*, *Tetrasphaera*, and *Zoogloea*) were widely distributed within various full-scale WWTPs (Zhang et al., 2012; Ju et al., 2014a; Saunders et al., 2016), indicating that functional bacteria may share a core community in different AS ecosystems. Aerobic heterotrophic bacteria were the most abundant functional genera, mainly responsible for the removal of organic matter (Du et al., 2020; Qiu et al., 2021). In the high-altitude and low-altitude groups, the proportion of aerobic heterotrophic bacteria was 25.75% ± 3.3% and 17.77% ± 2.3%, respectively (Figure 4B). This study identified two kinds of PAOs (*Tetrasphaera* and *Accumulibacter*) and one kind of GAOs (*Competibacter*). It has been reported that *Accumulibacter* alters its metabolic processes in the absence of intracellular polyphosphates, switching from PAO metabolism to GAOs metabolism (Welles et al., 2016). The relative abundance of PAOs in the high-altitude group (1.3% ± 0.8%) was higher than that in the low-altitude group (0.6% ± 0.3%) (*p* < 0.05), whereas the relative abundance of GAOs in the high-altitude group (0.4% ± 0.3%) was lower than that in the low-altitude group (2.8% ± 0.5%) (*p* < 0.01).

In this study, only *Nitrosomonas* of AOB was found, and the average relative abundances in high- and low-altitude groups were 1.5% ± 0.4% and 0.2% ± 0.1%, respectively. Meanwhile, only *Nitrospira* of NOB was found in this study, with a relative abundance of 0.8% ± 0.5% in the high-altitude group and 0.5% ± 0.2% in the low-altitude group. The total relative abundances of nine different genera of nitrite reduction bacteria (*Azoarcus*, *Dechloromonas*, *Denitratisoma*, *Haliangium*, *Nitrosomonas*, *Nitrospira*, *Rhodoferax*, *Sulfuritalea*, and *Zoogloea*) accounted for 7.9% ± 1.3% and 4.7% ± 0.9% at the high- and low-altitude groups, respectively. These results explained, to some extent, why there was no significant difference (*p* > 0.05) in the removal efficiency of nitrogen between high- and low-altitude WWTPs. The pH and DO of the biological system in the high-altitude group were controlled at 7.9 ± 0.3 and 2.8 ± 0.9 mg/L, respectively, which was within the suitable growth range of the bacterial population. These conditions were favorable to the growth of nitrogen and phosphorus-removing bacteria, thus probably increasing the microbial abundance of AS in WWTPs in this work.

Compared to Niu et al. (2016), a significant discrepancy was that the abundance of nitrogen and phosphorus removal bacteria was higher in the high-altitude WWTPs group. It might be due to the lower altitude (1,642 ± 106 m) and higher operational temperature (15.5°C ± 1.1°C) in this work. Moreover, different operational conditions, including pH and DO, would affect the functional bacterial population of AS in WWTPs. The pH and DO of the biological system in the high-altitude group were controlled at 7.9 ± 0.3 and 2.8 ± 0.9 mg/L, respectively, which were within the suitable growth range of the bacterial population. These conditions were favorable to the growth of nitrogen and phosphorus-removing bacteria, thus probably increasing the microbial abundance of AS in WWTPs in this work.

### 3.5 Correlation network analysis of dominant functional bacteria

**FIGURE 5:**
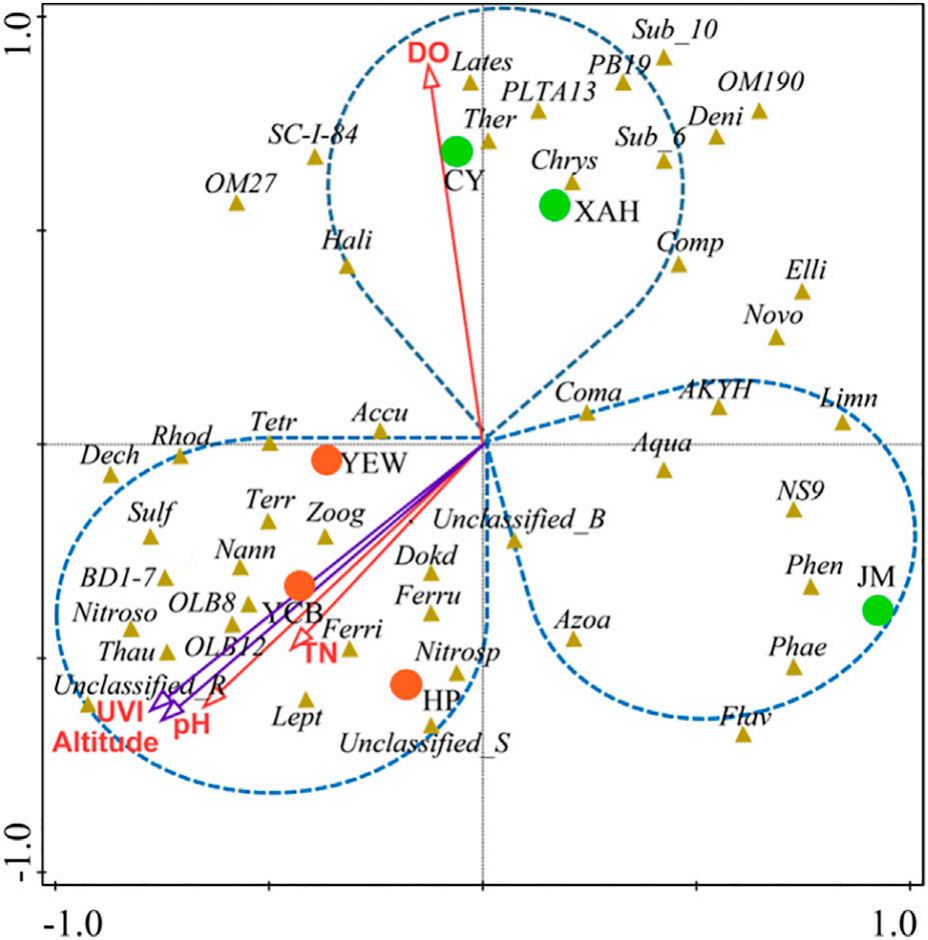
RDA of microbial community and environmental factors in all the samples. The environmental factors (altitude, DO, UVI, pH, and TN) that had a significant impact (*p* < 0.05) on the microbial community were screened out. The red circles represent high-altitude samples, and the green circles represent low-altitude samples (UVI, DO, and TN represent an ultraviolet index, dissolved oxygen, and total nitrogen, respectively, and the full names of bacteria are listed in Supplementary Table S6).

**FIGURE 6:**
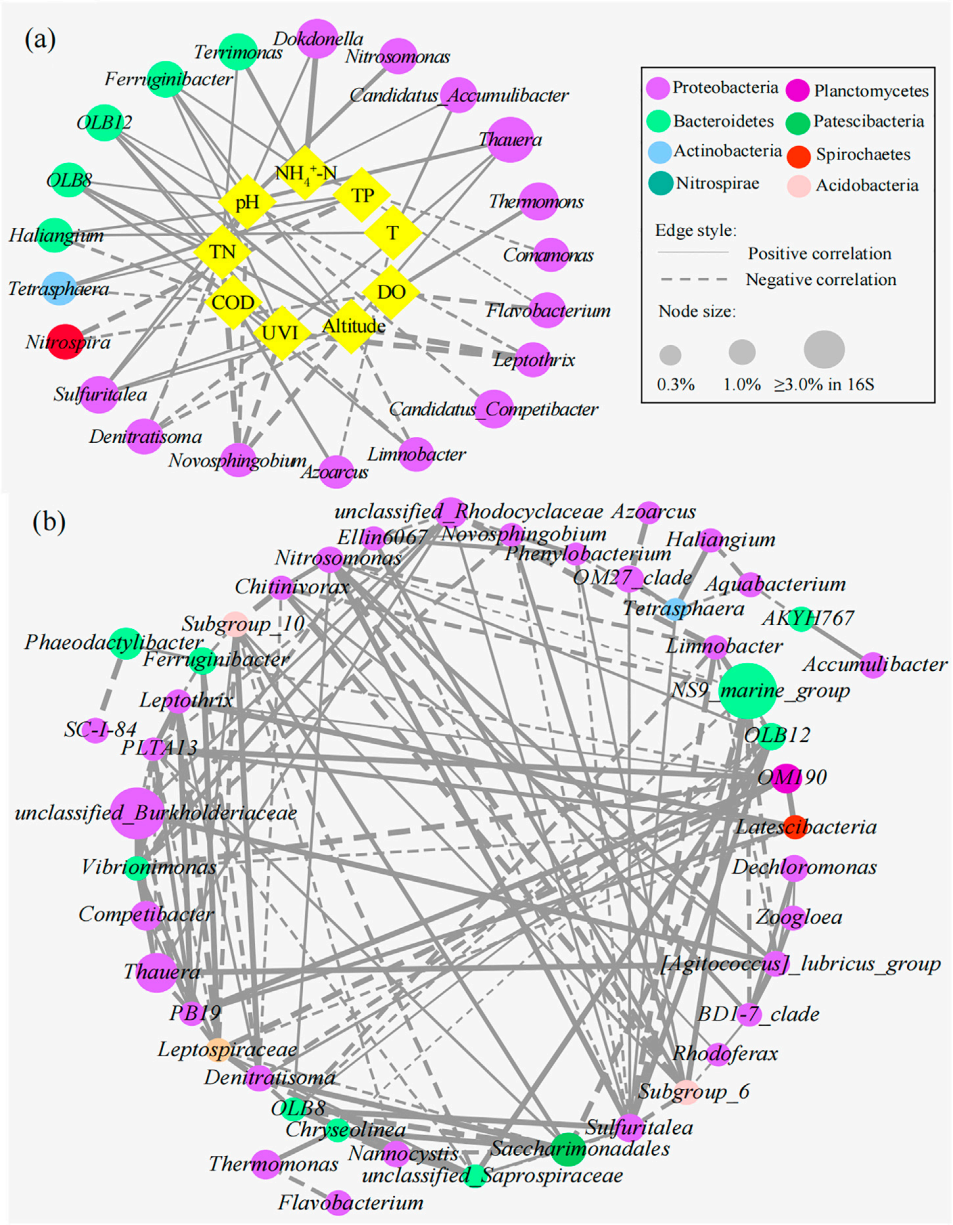
Correlation networks **(A)** between microbial communities and environmental variables and **(B)** within microorganisms at the genus level (the node size is proportional to its relative abundance. The higher the relative abundance, the larger the node. The solid line represents a positive correlation, the dotted line represents a negative correlation, and the thickness of the line represents the strength of the correlation. The thicker the line, the stronger the correlation.).

The interactions between specific microbial communities and environmental factors in WWTPs were explored by RDA and Spearman’s correlation analysis (Figure 5). Approximately nine variables, including environmental factors (altitude and UVI); operating parameters [temperature (T), pH, and DO]; and influent characteristics (influent COD, NH_4_^+^-N, TN, and TP), were selected. RDA showed that altitude, UVI, pH, DO, and TN were the strongest factors (*p* < 0.05) affecting the microbial communities, and these five variables explained 21.4%, 20.3%, 16.9%, 11.5%, and 8.2% of the bacterial assembly of AS communities, respectively. YEW, YCB, and HP were located in the third quadrant, closely related to altitude, UVI, and pH, indicating that their microbial communities were mainly affected by these three factors. CY and XAH were closely related to DO, which might be due to their higher DO (6.5 and 4.9 mg/L, respectively). For pH, the high-altitude group (7.62–8.12) was higher than the low-altitude group (6.81–7.20), and higher pH was conducive to the life activities of nitrogen- and phosphorus-removal bacteria (Oehmen et al., 2007; Park et al., 2010). On the contrary, the temperature did not become an important influencing factor. Supplementary Figures S3A, B show the correlation networks between the top 15 bacteria and environmental factors at the phylum and class level (Spearman’s |ρ| ≥ 0.65, *p* < 0.01). Figures 6A, B show the correlation networks between the functional genera (obtained by MiDAS analysis) and environmental variables and the correlation networks among the top 50 genera (Spearman’s |ρ| ≥ 0.65, *p* < 0.01). Altitude, UVI, and pH were most strongly correlated with microorganisms. According to the result of correlation networks, five phyla in 15 dominant phyla, seven classes in 15 dominant classes, and four genera in functional genera negatively correlated with altitude, suggesting that altitude influenced the relative abundance of some microorganisms. At the genus level, the relative abundance of *Thauera* (Spearman’s ρ = 0.71, *p* < 0.01), *Sulfuritalea* (Spearman’s ρ = 0.71, *p* < 0.01), and *Ferruginibacter* (Spearman’s ρ = 0.71, *p* < 0.01) were positively correlated with altitude. The relative abundance of four genera showed a strong negative correlation with UVI, for example, *Leptothrix* (Spearman’s ρ = −0.94, *p* < 0.01), *Novosphingobium* (Spearman’s ρ = −0.89, *p* < 0.01), and *Denitratisoma* (Spearman’s ρ = −0.77, *p* < 0.01). The relative abundance of some genera was positively correlated with pH, such as *Ferruginibacter* (Spearman’s ρ = 0.70, *p* < 0.01), *Nitrosomonas* (Spearman’s ρ = 0.84, *p* < 0.01), *Sulfuritalea* (Spearman’s ρ = 0.72, *p* < 0.01), and *Thauera* (Spearman’s ρ = 0.81, *p* < 0.01). Again, it was confirmed that temperature was not a key influencing factor. RDA and co-occurrence network indicated that TN in the influent characteristics was also an important factor affecting microbial composition. The relative abundance of some genera was positively correlated with TN, such as *Dokdonella* (Spearman’s ρ = 0.71, *p* < 0.01), *Sulfuritalea* (Spearman’s ρ = 0.71, *p* < 0.01), *Terrimonas* (Spearman’s ρ = 0.71, *p* < 0.01), and *Tetrasphaera* (Spearman’s ρ = 0.66, *p* < 0.01).

Supplementary Figure S4 shows the correlation network between the top 50 genera at the genus level and environmental factors in high- and low-altitude groups. The network centralization and density were higher in the high-altitude group (0.337 and 0.168, respectively) than those in the low-altitude group (0.163 and 0.150, respectively), indicating that the high-altitude symbiotic network had a better modular structure and was more closely connected to the environment. The less the heterogeneity in the network, the higher the robustness and sensitivity (Berry and Widder, 2014). The heterogeneity in the high-altitude group (0.608) was lower than that in the low-altitude group (0.611), indicating higher sensitivity in the high-altitude group. The characteristic of industrial wastewater can greatly affect the microbial community composition (Gao et al., 2016). Due to the higher proportion of industrial wastewater (60%) in influent of CY WWTP, its microbial diversity and complexity were lower than the other two low-altitude WWTPs. This was consistent with the results obtained by Ouyang et al. (2019), who found that some toxic substances in industrial wastewater could reduce microbial community diversity. The correlation analysis between microorganisms and environmental factors indicated that high-altitude and strong UVI were unfavorable to the survival of most bacteria in AS. Therefore, in order to optimize the operation of WWTPs in high-altitude regions, the influence of UVI on the open-air WWTPs should be seriously considered during the design and operation of WWTP to maximize the effectiveness in biological nutrient removal.

Based on microbial abundance data, ecological networks among genera were constructed, and then interactions among genera were inferred (Hibbing et al., 2010; Molloy, 2014; Fuhrman et al., 2015). The genus-level microbial correlation network was a complex network of community relationships consisting of 47 bacterial nodes and 143 edges (Figure 6B; Supplementary Table S7). The structure of the co-occurrence network included an average path length of 4.3, a modularity index of 0.44, and a network diameter of 6. The modularity index >0.4 suggested that the network had a modular structure. Most nodes had a low degree, and only a few core nodes had a high degree, indicating that the network had a non-random coexistence pattern. Interestingly, genera with high vertex centrality were *Sulfuritalea*, *Vibrionimonas*, *PLTA13*, *Leptospiraceae*, and *unclassified_Saprospiraceae*. This was consistent with previous studies that species with less abundance played an important role in the interaction correlation network (Ju et al., 2014b; Xia et al., 2016). The positive correlation of *Nitrosomonas* and *Sulfuritalea* (Spearman’s ρ = 0.99, *p* < 0.01), *Zoogloea*, and *Dechloromonas* (Spearman’s ρ = 0.83, *p* < 0.01) may reflect the synergistic effect in the nitrogen removal process. The positive correlation of *Saccharimonadales* and *Denitratisoma* (Spearman’s ρ = 0.94, *p* < 0.01) embodied a mutualistic symbiosis relationship, and the former could degrade large organic molecules (e.g., protein hydrolysis) into small organic molecules, which were readily available to the latter. There were negative correlations between *Tetrasphaera* and *Aquabacterium* (Spearman’s ρ = −0.94, *p* < 0.01) and *Saccharimonadales* and the *unclassified_Saprospiraceae* (Spearman’s ρ = −0.93, *p* < 0.01). Among them, *Tetrasphaera* is a PAOs that uses organic matter as a carbon source to remove phosphorus through a fermentation pathway (Zhao et al., 2022); *Aquabacterium* belongs to denitrifying bacteria, and the content of organic carbon source directly determines its denitrification performance (Zhang et al., 2022); *Saccharimonadales* can hydrolyze organic P, but not without an organic carbon source (Wang et al., 2022); and *unclassified_Saprospiraceae* play a crucial role in the degradation of organic compounds in AS processes (Carluccio et al., 2023). The metabolic processes of these four bacteria are inseparable from organic matter, so the negative correlation between them reflects the competitive role in organic matter utilization. The phylogenetic relationships of 16S rRNA in bacterial communities at the genus level are shown in Supplementary Figure S5. The phylogenetic relationship of bacterial communities with a positive correlation was closer than that with a negative correlation probably because they had similar niche preferences or tended to cooperate (such as mutualism) (Losos, 2008; Philippot et al., 2010; Ju and Zhang, 2015).

## 4 Conclusion

There were significant differences in bacterial communities of AS samples between high- and low-altitude regions. Microbial diversities and richness in the high-altitude group were lower than those in the low-altitude group. A unique microbial community structure was observed in the high-altitude region. The combination of RDA and co-occurrence network analysis showed that altitude, UVI, pH, DO, and TN were the predominant environmental factors affecting microbial community assembly in the AS system of WWTPs. However, the temperature was not a limiting element in this study. Furthermore, low temperature and strong UVI are the most pivotal factors that influence the microbial diversities and complexity of AS microbial community in high-altitude regions. Further research is needed to optimize the microbial community structure by adjusting UVI for the open-air wastewater biological treatment system.

## Data Availability

The original contributions presented in the study are publicly available. This data can be found here: https://dataview.ncbi.nlm.nih.gov/object/PRJNA936450.
