# Supplementary material for: Deciphering the microbial community structures and functions of wastewater treatment at high-altitude area
Source: Front Bioeng Biotechnol. 2023 Feb 27;11:1107633. doi: 10.3389/fbioe.2023.1107633 (PMC10009103; doi:10.3389/fbioe.2023.1107633)
Supplement: Supplementary file 1 [file DataSheet1.docx]

***Supplementary materials***

**Figure S1** Rarefaction Curve of all samples.

**Figure S2** The shared core OTUs (a) and percentage and relative abundance (b) relative to the whole OTUs

**Figure S3** Correlation network between microbial community and environmental variables at (a) phylum and (b) class level

**Figure S4** Correlation network between bacteria and environmental factors at genus level of (a) high-altitude and (b) low-altitude, respectively

**Figure S5** Taxonomic rank tree of species with abundance information

**Table S1** The treatment performance of six WWTPs

**Table S2** Richness and diversity of microbial community in activated sludge for six municipal WWTPs

**Table S3** The dominant phyla in each WWTP (%)

**Table S4** The dominant class in each WWTP (%)

**Table S5** The composition of the core OTUs

**Table S6** Abbreviation and full name of bacteria in RDA diagram

**Table S7** Spearman's correlation coefficient (ρ) at genus level

**Figure S1** Rarefaction Curve of all samples.


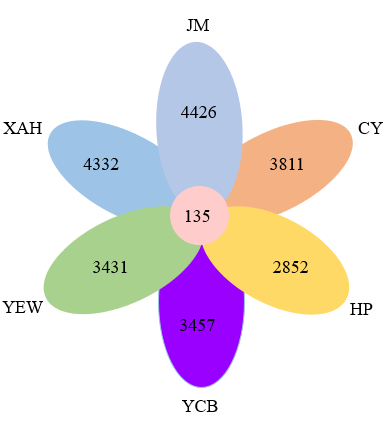


(b)

(a)

**Figure S2** The shared core OTUs (a) and percentage and relative abundance (b) relative to the whole OTUs

(a)

(b)

**Figure S3** Correlation network between microbial community and environmental variables at (a) phylum and (b) class level


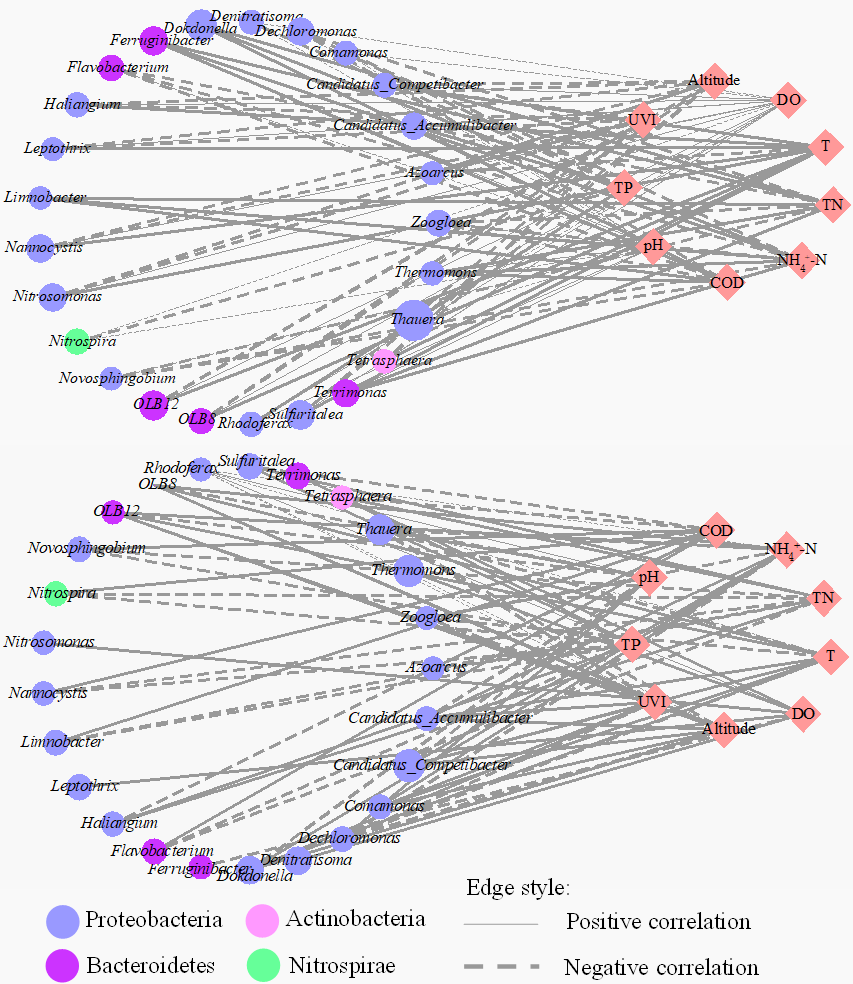


**Figure S4** Correlation network between bacteria and environmental factors at genus level of (a) high-altitude and (b) low-altitude, respectively

**Figure S5** Taxonomic rank tree of species with abundance information

**Table S1** The treatment performance of six WWTPs

| Group | Sample | Influent (mg/L) | | | | Effluent (mg/L) | | | | Removal efficiency (%) | | | |
| --- | --- | --- | --- | --- | --- | --- | --- | --- | --- | --- | --- | --- | --- |
|  |  | COD | NH_4_^+^-N | TN | TP | COD | NH_4_^+^-N | TN | TP | COD | NH_4_^+^-N | TN | TP |
| High-altitude | YEW | 218 | 58.8 | 60.1 | 8.7 | 19 | 0.1 | 11.8 | 0.3 | 91.3 | 99.8 | 80.3 | 96.6 |
|  | YCB | 106 | 47.6 | 53.1 | 1.8 | 19 | 0.5 | 15.9 | 0.1 | 82.1 | 93.9 | 70.0 | 94.4 |
|  | HP | 534 | 85.3 | 101.0 | 2.9 | 133 | 3.6 | 17.6 | 0.1 | 75.1 | 95.8 | 82.6 | 96.6 |
| Low-altitude | XAH | 155 | 41.6 | 59.6 | 5.6 | 16 | 0.6 | 8.1 | 0.2 | 89.7 | 98.5 | 86.4 | 96.4 |
|  | CY | 266 | 37.9 | 47.0 | 5.1 | 49 | 0.1 | 9.6 | 0.2 | 77.8 | 99.7 | 81.6 | 96.0 |
|  | JM | 954 | 39.1 | 46.7 | 1.7 | 209 | 0.2 | 5.7 | 0.1 | 78.1 | 99.5 | 87.8 | 94.1 |

**Table S2** Richness and diversity of microbial community in activated sludge for six municipal WWTPs

| Group | Sample | Sequences | OTUs | Chao1 | Shannon | Simpson | Good's coverage |
| --- | --- | --- | --- | --- | --- | --- | --- |
| High-altitude | YEW | 42466 | 3566 | 2094 | 9.08 | 0.996 | 0.987 |
|  | YCB | 55554 | 3592 | 2413 | 9.11 | 0.994 | 0.983 |
|  | HP | 45616 | 2987 | 1848 | 8.27 | 0.989 | 0.989 |
| Low-altitude | XAH | 60450 | 4447 | 3000 | 9.38 | 0.994 | 0.992 |
|  | CY | 65148 | 3946 | 2783 | 9.10 | 0.992 | 0.992 |
|  | JM | 57815 | 4461 | 2984 | 8.84 | 0.985 | 0.993 |

**Table S3** The dominant phyla in each WWTP (%)

| Taxon | High-altitude | | | low-altitude | | |
| --- | --- | --- | --- | --- | --- | --- |
|  | YEW | HP | YCB | XAH | JM | CY |
| *Proteobacteria* | 48.60 | 50.38 | 56.96 | 49.27 | 41.19 | 47.94 |
| *Bacteroidetes* | 36.53 | 32.14 | 25.77 | 21.34 | 41.42 | 16.78 |
| *Planctomycetes* | 2.91 | 3.93 | 2.88 | 6.34 | 6.39 | 7.49 |
| *Chloroflexi* | 3.04 | 5.94 | 4.98 | 6.14 | 1.97 | 1.84 |
| *Acidobacteria* | 0.85 | 0.92 | 2.26 | 5.67 | 2.27 | 5.32 |
| *Patescibacteria* | 0.93 | 0.70 | 0.75 | 3.01 | 0.78 | 10.98 |
| *Actinobacteria* | 1.20 | 1.16 | 0.64 | 2.87 | 1.42 | 1.92 |
| *Verrucomicrobia* | 0.58 | 0.21 | 0.34 | 1.10 | 0.34 | 0.25 |
| *Gemmatimonadetes* | 0.44 | 0.37 | 0.25 | 1.06 | 1.29 | 2.32 |
| *Armatimonadetes* | 0.19 | 0.08 | 0.12 | 0.62 | 0.21 | 0.07 |
| Others | 4.72 | 4.18 | 5.06 | 2.59 | 2.73 | 5.10 |

**Table S4** The dominant class in each WWTP (%)

| Taxon | High-altitude | | | low-altitude | | |
| --- | --- | --- | --- | --- | --- | --- |
|  | YEW | HP | YCB | XAH | JM | CY |
| *Gammaproteobacteria* | 36.61 | 42.85 | 45.25 | 35.74 | 30.06 | 39.31 |
| *Bacteroidia* | 33.64 | 31.09 | 24.35 | 19.83 | 40.78 | 15.32 |
| *Alphaproteobacteria* | 2.79 | 2.57 | 3.90 | 7.68 | 7.51 | 3.79 |
| *Deltaproteobacteria* | 9.13 | 4.75 | 7.76 | 5.81 | 3.58 | 4.82 |
| *Anaerolineae* | 2.83 | 5.41 | 4.57 | 5.51 | 1.36 | 1.31 |
| *OM190* | 1.31 | 1.91 | 0.97 | 3.53 | 2.30 | 3.69 |
| *Saccharimonadia* | 0.69 | 0.61 | 0.62 | 2.18 | 0.62 | 10.26 |
| *Acidimicrobiia* | 0.49 | 0.66 | 0.42 | 2.12 | 0.88 | 1.55 |
| *Thermoanaerobaculia* | 0.04 | 0.07 | 0.18 | 2.00 | 0.19 | 1.98 |
| *Subgroup 4* | 0.09 | 0.43 | 0.54 | 1.99 | 0.35 | 0.87 |
| others | 12.39 | 9.64 | 11.46 | 13.61 | 12.39 | 17.11 |

**Table S5** The composition of the core OTUs

| Number of OTUs | Abundance of 16S rRNA (%) | | | | | | Ecological classification | | | |
| --- | --- | --- | --- | --- | --- | --- | --- | --- | --- | --- |
|  | XAH | JM | CY | YEW | HP | YCB | Phylum | Class | Family | Genus |
| 1 | 0.21 | 0.20 | 1.60 | 0.28 | 0.60 | 0.35 | *Proteobacteria* | *Gammaproteobacteria* | *Burkholderiaceae* | *Leptothrix* |
| 2 | 0.23 | 0.08 | 0.81 | 0.13 | 0.07 | 0.06 | *Proteobacteria* | *Gammaproteobacteria* | *PLTA13* | *PLTA13* |
| 3 | 0.70 | 0.51 | 0.65 | 0.04 | 0.03 | 0.17 | *Proteobacteria* | *Gammaproteobacteria* | *Rhodocyclaceae* | *Denitratisoma* |
| 4 | 2.36 | 0.97 | 0.62 | 1.85 | 4.95 | 0.07 | *Proteobacteria* | *Gammaproteobacteria* | *Rhodanobacteraceae* | *Dokdonella* |
| 5 | 0.71 | 0.88 | 1.71 | 2.07 | 1.22 | 4.27 | *Proteobacteria* | *Gammaproteobacteria* | *Rhodocyclaceae* | *Thauera* |
| 6 | 0.28 | 0.01 | 1.02 | 0.03 | 0.29 | 0.00 | *Patescibacteria* | *Saccharimonadia* | *Saccharimonadales* | *Saccharimonadales* |
| 7 | 0.37 | 1.25 | 0.54 | 0.08 | 0.44 | 0.08 | *Bacteroidetes* | *Bacteroidia* | *NS9_marine_group* | *NS9_marine_group* |
| 8 | 0.24 | 0.25 | 0.39 | 0.34 | 0.43 | 0.63 | *Proteobacteria* | *Gammaproteobacteria* | *SC-I-84* | *SC-I-84* |
| 9 | 0.04 | 0.39 | 0.39 | 0.36 | 0.44 | 1.15 | *Nitrospirae* | *Nitrospira* | *Nitrospiraceae* | *Nitrospira* |
| 10 | 0.13 | 0.32 | 0.16 | 0.04 | 0.02 | 0.20 | *Proteobacteria* | *Gammaproteobacteria* | *Nitrosomonadaceae* | *Ellin6067* |
| 11 | 0.11 | 0.51 | 0.63 | 0.30 | 0.42 | 0.53 | *Proteobacteria* | *Gammaproteobacteria* | *Burkholderiaceae* | *Aquabacterium* |
| 12 | 0.51 | 0.14 | 0.18 | 0.06 | 0.04 | 0.05 | *Proteobacteria* | *Gammaproteobacteria* | *Burkholderiaceae* | *unclassified_Burkholderiaceae* |
| 13 | 0.71 | 0.37 | 0.47 | 0.41 | 2.65 | 0.77 | *Proteobacteria* | *Gammaproteobacteria* | *Rhodocyclaceae* | *Thauera* |
| 14 | 0.08 | 0.03 | 0.20 | 0.02 | 0.05 | 0.02 | *Proteobacteria* | *Gammaproteobacteria* | *Burkholderiaceae* | *Limnobacter* |
| 15 | 0.66 | 0.25 | 0.58 | 0.39 | 1.03 | 0.14 | *Proteobacteria* | *Gammaproteobacteria* | *Burkholderiaceae* | *unclassified_Burkholderiaceae* |
| 16 | 0.06 | 0.05 | 0.07 | 0.05 | 0.05 | 0.04 | *Firmicutes* | *Bacilli* | *Carnobacteriaceae* | *Trichococcus* |
| 17 | 0.21 | 0.39 | 0.42 | 0.10 | 0.14 | 0.07 | *Bacteroidetes* | *Bacteroidia* | *env.OPS_17* | *env.OPS_17* |
| 18 | 0.06 | 0.07 | 0.15 | 0.06 | 0.05 | 0.06 | *Proteobacteria* | *Gammaproteobacteria* | *Aeromonadaceae* | *Aeromonas* |
| 19 | 0.13 | 0.20 | 0.30 | 0.29 | 0.06 | 0.36 | *Proteobacteria* | *Gammaproteobacteria* | *Halomonadaceae* | *Halomonas* |
| 20 | 0.07 | 0.03 | 0.07 | 0.04 | 0.05 | 0.03 | *Bacteroidetes* | *Bacteroidia* | *Prevotellaceae* | *Prevotella_9* |
| 21 | 0.04 | 0.14 | 0.18 | 0.23 | 0.38 | 0.47 | *Proteobacteria* | *Gammaproteobacteria* | *Burkholderiaceae* | *unclassified_Burkholderiaceae* |
| 22 | 0.39 | 0.11 | 0.27 | 0.79 | 0.21 | 0.80 | *Proteobacteria* | *Gammaproteobacteria* | *Rhodocyclaceae* | *Sulfuritalea* |
| 23 | 0.07 | 0.22 | 0.62 | 0.07 | 0.05 | 0.03 | *Bacteroidetes* | *Bacteroidia* | *env.OPS_17* | *env.OPS_17* |
| 24 | 0.22 | 0.35 | 0.40 | 0.37 | 0.58 | 0.56 | *Proteobacteria* | *Gammaproteobacteria* | *Chitinimonadaceae* | *Chitinivorax* |
| 25 | 0.17 | 0.13 | 0.24 | 0.28 | 0.34 | 0.36 | *Proteobacteria* | *Gammaproteobacteria* | *Burkholderiaceae* | *unclassified_Burkholderiaceae* |
| 26 | 0.08 | 0.17 | 0.28 | 0.05 | 0.02 | 0.18 | *Proteobacteria* | *Alphaproteobacteria* | *Sphingomonadaceae* | *uncultured* |
| 27 | 0.08 | 0.14 | 0.16 | 0.14 | 0.24 | 0.44 | *Proteobacteria* | *Gammaproteobacteria* | *Burkholderiaceae* | *Aquabacterium* |
| 28 | 0.46 | 0.01 | 0.27 | 0.95 | 0.02 | 0.80 | *Proteobacteria* | *Deltaproteobacteria* | *Bdellovibrionaceae* | *OM27_clade* |
| 29 | 0.07 | 0.03 | 0.34 | 0.11 | 0.07 | 0.09 | *Patescibacteria* | *Saccharimonadia* | *Saccharimonadales* | *Saccharimonadales* |
| 30 | 0.08 | 0.10 | 0.32 | 0.15 | 0.09 | 0.88 | *Proteobacteria* | *Gammaproteobacteria* | *Rhodocyclaceae* | *Thauera* |
| 31 | 0.14 | 0.20 | 0.45 | 0.01 | 0.02 | 0.06 | *Proteobacteria* | *Gammaproteobacteria* | *Hydrogenophilaceae* | *uncultured* |
| 32 | 0.04 | 0.09 | 0.12 | 0.09 | 0.18 | 0.20 | *Acidobacteria* | *Subgroup_6* | *Subgroup_6* | *Subgroup_6* |
| 33 | 0.07 | 0.05 | 0.11 | 1.67 | 0.07 | 0.90 | *Proteobacteria* | *Deltaproteobacteria* | *Nannocystaceae* | *Nannocystis* |
| 34 | 0.03 | 0.00 | 0.05 | 0.04 | 0.03 | 0.02 | *Bacteroidetes* | *Bacteroidia* | *Prevotellaceae* | *Prevotella_9* |
| 35 | 0.28 | 0.06 | 0.12 | 0.08 | 0.10 | 0.04 | *Proteobacteria* | *Gammaproteobacteria* | *unclassified_Gammaproteobacteria* | *unclassified_Gammaproteobacteria* |
| 36 | 0.02 | 0.13 | 0.19 | 0.05 | 0.06 | 0.16 | *Proteobacteria* | *Gammaproteobacteria* | *Burkholderiaceae* | *unclassified_Burkholderiaceae* |
| 37 | 0.08 | 0.04 | 0.31 | 0.10 | 0.02 | 0.12 | *Bacteroidetes* | *Bacteroidia* | *NS11-12_marine_group* | *NS11-12_marine_group* |
| 38 | 0.02 | 0.10 | 0.17 | 0.09 | 0.13 | 0.12 | *Proteobacteria* | *Gammaproteobacteria* | *Rhodocyclaceae* | *Sterolibacterium* |
| 39 | 0.04 | 0.26 | 0.13 | 0.01 | 0.14 | 0.29 | *Nitrospirae* | *Nitrospira* | *Nitrospiraceae* | *Nitrospira* |
| 40 | 0.19 | 0.09 | 0.12 | 0.03 | 0.12 | 0.13 | *Proteobacteria* | *Gammaproteobacteria* | *CCM19a* | *CCM19a* |
| 41 | 0.03 | 0.02 | 0.08 | 0.04 | 0.04 | 0.05 | *Bacteroidetes* | *Bacteroidia* | *Tannerellaceae* | *Macellibacteroides* |
| 42 | 0.05 | 0.02 | 0.04 | 0.02 | 0.01 | 0.02 | *Bacteroidetes* | *Bacteroidia* | *Bacteroidaceae* | *Bacteroides* |
| 43 | 0.13 | 0.15 | 0.09 | 0.06 | 0.03 | 0.11 | *Planctomycetes* | *Planctomycetacia* | *Rubinisphaeraceae* | *uncultured* |
| 44 | 0.03 | 0.11 | 0.18 | 0.06 | 0.04 | 0.49 | *Proteobacteria* | *Gammaproteobacteria* | *Rhodocyclaceae* | *Zoogloea* |
| 45 | 0.02 | 0.01 | 0.05 | 0.00 | 0.02 | 0.01 | *Firmicutes* | *Bacilli* | *Streptococcaceae* | *Lactococcus* |
| 46 | 0.42 | 0.28 | 0.10 | 0.07 | 0.12 | 0.13 | *Verrucomicrobia* | *Verrucomicrobiae* | *Opitutaceae* | *Cephaloticoccus* |
| 47 | 0.03 | 0.03 | 0.04 | 0.02 | 0.05 | 0.02 | *Planctomycetes* | *Phycisphaerae* | *Phycisphaeraceae* | *SM1A02* |
| 48 | 0.02 | 0.01 | 0.07 | 0.04 | 0.01 | 0.02 | *Fusobacteria* | *Fusobacteriia* | *Leptotrichiaceae* | *Hypnocyclicus* |
| 49 | 0.01 | 0.11 | 0.09 | 0.02 | 0.12 | 0.17 | *Proteobacteria* | *Gammaproteobacteria* | *Burkholderiaceae* | *Acidovorax* |
| 50 | 0.01 | 0.01 | 0.02 | 0.00 | 0.00 | 0.01 | *Bacteroidetes* | *Bacteroidia* | *Bacteroidaceae* | *Bacteroides* |
| 51 | 0.03 | 0.08 | 0.10 | 0.08 | 0.12 | 0.20 | *Epsilonbacteraeota* | *Campylobacteria* | *Arcobacteraceae* | *Arcobacter* |
| 52 | 0.67 | 0.08 | 0.10 | 0.30 | 0.04 | 0.26 | *Proteobacteria* | *Gammaproteobacteria* | *Rhodanobacteraceae* | *uncultured* |
| 53 | 0.08 | 0.05 | 0.09 | 0.47 | 0.09 | 0.23 | *Bacteroidetes* | *Ignavibacteria* | *PHOS-HE36* | *PHOS-HE36* |
| 54 | 0.04 | 0.06 | 0.04 | 0.06 | 0.16 | 0.15 | *Proteobacteria* | *Gammaproteobacteria* | *Moraxellaceae* | *Acinetobacter* |
| 55 | 0.11 | 0.07 | 0.10 | 0.04 | 0.19 | 0.07 | *Proteobacteria* | *Alphaproteobacteria* | *Rhizobiales_Incertae_Sedis* | *uncultured* |
| 56 | 0.02 | 0.02 | 0.06 | 0.02 | 0.01 | 0.03 | *Proteobacteria* | *Gammaproteobacteria* | *Steroidobacteraceae* | *uncultured* |
| 57 | 0.08 | 0.10 | 0.13 | 0.07 | 0.39 | 0.41 | *Proteobacteria* | *Gammaproteobacteria* | *Moraxellaceae* | *[Agitococcus]_lubricus_group* |
| 58 | 0.07 | 0.06 | 0.05 | 0.17 | 0.06 | 0.02 | *Proteobacteria* | *Deltaproteobacteria* | *mle1-27* | *mle1-27* |
| 59 | 0.03 | 0.02 | 0.10 | 1.20 | 0.51 | 0.59 | *Bacteroidetes* | *Bacteroidia* | *Saprospiraceae* | *uncultured* |
| 60 | 0.31 | 0.05 | 0.08 | 0.09 | 0.08 | 0.02 | *Proteobacteria* | *Gammaproteobacteria* | *Rhodocyclaceae* | *Sulfuritalea* |
| 61 | 0.16 | 0.04 | 0.06 | 0.13 | 0.04 | 0.13 | *Acidobacteria* | *Subgroup_17* | *Subgroup_17* | *Subgroup_17* |
| 62 | 0.25 | 0.08 | 0.10 | 0.21 | 0.37 | 0.11 | *Proteobacteria* | *Deltaproteobacteria* | *mle1-27* | *mle1-27* |
| 63 | 0.02 | 0.05 | 0.10 | 0.10 | 0.09 | 0.12 | *Bacteroidetes* | *Bacteroidia* | *KD3-93* | *KD3-93* |
| 64 | 0.08 | 0.13 | 0.09 | 0.14 | 0.09 | 0.22 | *Proteobacteria* | *Gammaproteobacteria* | *Rhodocyclaceae* | *Dechloromonas* |
| 65 | 0.01 | 0.04 | 0.05 | 0.08 | 0.03 | 0.23 | *Proteobacteria* | *Gammaproteobacteria* | *Burkholderiaceae* | *unclassified_Burkholderiaceae* |
| 66 | 0.04 | 0.06 | 0.07 | 0.40 | 0.07 | 0.07 | *Proteobacteria* | *Gammaproteobacteria* | *Chitinimonadaceae* | *Chitinivorax* |
| 67 | 0.04 | 0.02 | 0.08 | 0.37 | 0.51 | 0.08 | *Bacteroidetes* | *Bacteroidia* | *Saprospiraceae* | *uncultured* |
| 68 | 0.16 | 0.18 | 0.05 | 0.07 | 0.22 | 0.17 | *Planctomycetes* | *Planctomycetacia* | *Pirellulaceae* | *Pirellula* |
| 69 | 0.08 | 0.09 | 0.03 | 0.04 | 0.01 | 0.12 | *Proteobacteria* | *Gammaproteobacteria* | *Burkholderiaceae* | *Ramlibacter* |
| 70 | 0.03 | 0.08 | 0.03 | 0.06 | 0.04 | 0.03 | *Proteobacteria* | *Gammaproteobacteria* | *PLTA13* | *PLTA13* |
| 71 | 0.02 | 0.07 | 0.06 | 0.01 | 0.04 | 0.04 | *Proteobacteria* | *Alphaproteobacteria* | *Sphingomonadaceae* | *Novosphingobium* |
| 72 | 0.02 | 0.02 | 0.05 | 0.02 | 0.01 | 0.03 | *Proteobacteria* | *Gammaproteobacteria* | *Xanthomonadaceae* | *Pseudoxanthomonas* |
| 73 | 0.01 | 0.11 | 0.08 | 0.22 | 0.23 | 0.23 | *Bacteroidetes* | *Bacteroidia* | *KD3-93* | *KD3-93* |
| 74 | 0.02 | 0.02 | 0.04 | 0.08 | 0.11 | 0.21 | *Kiritimatiellaeota* | *Kiritimatiellae* | *Kiritimatiellae* | *Kiritimatiellae* |
| 75 | 0.02 | 0.01 | 0.02 | 0.03 | 0.04 | 0.01 | *Proteobacteria* | *Gammaproteobacteria* | *Unknown_Family* | *Acidibacter* |
| 76 | 0.01 | 0.04 | 0.04 | 0.01 | 0.03 | 0.02 | *Bacteroidetes* | *Bacteroidia* | *NS9_marine_group* | *NS9_marine_group* |
| 77 | 0.15 | 0.15 | 0.11 | 0.46 | 0.16 | 0.02 | *Proteobacteria* | *Gammaproteobacteria* | *Rhodocyclaceae* | *Candidatus_Accumulibacter* |
| 78 | 0.01 | 0.11 | 0.06 | 0.18 | 0.12 | 0.09 | *Bacteroidetes* | *Bacteroidia* | *Crocinitomicaceae* | *Crocinitomix* |
| 79 | 0.04 | 0.02 | 0.04 | 0.02 | 0.02 | 0.15 | *Acidobacteria* | *Acidobacteriia* | *Solibacteraceae_(Subgroup_3)* | *Bryobacter* |
| 80 | 0.04 | 0.07 | 0.08 | 0.46 | 0.82 | 0.46 | *Bacteroidetes* | *Bacteroidia* | *Saprospiraceae* | *uncultured* |
| 81 | 0.00 | 0.00 | 0.04 | 0.00 | 0.04 | 0.01 | *Proteobacteria* | *Gammaproteobacteria* | *Burkholderiaceae* | *Leptothrix* |
| 82 | 0.07 | 0.11 | 0.07 | 0.07 | 0.09 | 0.09 | *Bacteroidetes* | *Bacteroidia* | *37-13* | *37-13* |
| 83 | 0.03 | 0.04 | 0.10 | 0.38 | 0.03 | 0.69 | *Bacteroidetes* | *Bacteroidia* | *uncultured* | *uncultured* |
| 84 | 0.01 | 0.02 | 0.02 | 0.08 | 0.02 | 0.06 | *Epsilonbacteraeota* | *Campylobacteria* | *Arcobacteraceae* | *Arcobacter* |
| 85 | 0.01 | 0.03 | 0.07 | 0.03 | 0.03 | 0.00 | *Bacteroidetes* | *Bacteroidia* | *Crocinitomicaceae* | *Fluviicola* |
| 86 | 0.05 | 0.09 | 0.03 | 0.01 | 0.05 | 0.00 | *Proteobacteria* | *Deltaproteobacteria* | *KD3-10* | *KD3-10* |
| 87 | 0.12 | 0.21 | 0.04 | 0.20 | 0.10 | 0.12 | *Proteobacteria* | *Gammaproteobacteria* | *Nitrosomonadaceae* | *oc32* |
| 88 | 0.01 | 0.00 | 0.02 | 0.07 | 0.03 | 0.02 | *Firmicutes* | *Clostridia* | *Eubacteriaceae* | *uncultured* |
| 89 | 0.02 | 0.02 | 0.03 | 0.04 | 0.05 | 0.06 | *Bacteroidetes* | *Bacteroidia* | *Bacteroidetes_vadinHA17* | *Bacteroidetes_vadinHA17* |
| 90 | 0.01 | 0.05 | 0.03 | 0.01 | 0.01 | 0.04 | *Proteobacteria* | *Gammaproteobacteria* | *Burkholderiaceae* | *unclassified_Burkholderiaceae* |
| 91 | 0.02 | 0.03 | 0.07 | 0.01 | 0.03 | 0.07 | *Bacteroidetes* | *Bacteroidia* | *Cryomorphaceae* | *uncultured* |
| 92 | 0.01 | 0.05 | 0.04 | 0.08 | 0.17 | 0.16 | *Proteobacteria* | *Gammaproteobacteria* | *Rhodocyclaceae* | *Thauera* |
| 93 | 0.01 | 0.03 | 0.05 | 0.01 | 0.02 | 0.05 | *Proteobacteria* | *Gammaproteobacteria* | *unclassified_Gammaproteobacteria* | *unclassified_Gammaproteobacteria* |
| 94 | 0.00 | 0.03 | 0.03 | 0.00 | 0.01 | 0.02 | *Proteobacteria* | *Gammaproteobacteria* | *Burkholderiaceae* | *unclassified_Burkholderiaceae* |
| 95 | 0.13 | 0.03 | 0.03 | 0.12 | 0.04 | 0.04 | *Proteobacteria* | *Deltaproteobacteria* | *PB19* | *PB19* |
| 96 | 0.00 | 0.00 | 0.03 | 0.01 | 0.01 | 0.61 | *Proteobacteria* | *Gammaproteobacteria* | *Rhodocyclaceae* | *Zoogloea* |
| 97 | 0.09 | 0.23 | 0.03 | 0.02 | 0.06 | 0.05 | *Bacteroidetes* | *Bacteroidia* | *uncultured* | *uncultured* |
| 98 | 0.00 | 0.01 | 0.04 | 0.05 | 0.00 | 0.04 | *Acidobacteria* | *Aminicenantia* | *Aminicenantales* | *Aminicenantales* |
| 99 | 0.10 | 0.00 | 0.01 | 0.02 | 0.06 | 0.06 | *Actinobacteria* | *Acidimicrobiia* | *Ilumatobacteraceae* | *CL500-29_marine_group* |
| 100 | 0.02 | 0.00 | 0.03 | 0.12 | 0.05 | 0.05 | *Bacteroidetes* | *Bacteroidia* | *AKYH767* | *AKYH767* |
| 101 | 0.00 | 0.00 | 0.01 | 0.01 | 0.00 | 0.01 | *Proteobacteria* | *Alphaproteobacteria* | *Acetobacteraceae* | *Roseomonas* |
| 102 | 0.14 | 0.26 | 0.03 | 0.10 | 0.04 | 0.04 | *Proteobacteria* | *Alphaproteobacteria* | *Sphingomonadaceae* | *unclassified_Sphingomonadaceae* |
| 103 | 0.01 | 0.03 | 0.04 | 0.20 | 0.00 | 0.05 | *Proteobacteria* | *Gammaproteobacteria* | *unclassified_Gammaproteobacteria* | *unclassified_Gammaproteobacteria* |
| 104 | 0.02 | 0.01 | 0.01 | 0.43 | 0.03 | 0.34 | *Proteobacteria* | *Deltaproteobacteria* | *Nannocystaceae* | *Nannocystis* |
| 105 | 0.00 | 0.03 | 0.01 | 0.02 | 0.00 | 0.01 | *Proteobacteria* | *Gammaproteobacteria* | *TRA3-20* | *TRA3-20* |
| 106 | 0.02 | 0.24 | 0.02 | 0.16 | 0.22 | 0.16 | *Proteobacteria* | *Gammaproteobacteria* | *Burkholderiaceae* | *Lautropia* |
| 107 | 0.01 | 0.06 | 0.01 | 0.03 | 0.01 | 0.03 | *Proteobacteria* | *Deltaproteobacteria* | *Bradymonadales* | *Bradymonadales* |
| 108 | 0.12 | 0.11 | 0.01 | 0.06 | 0.05 | 0.02 | *Proteobacteria* | *Gammaproteobacteria* | *Burkholderiaceae* | *Limnobacter* |
| 109 | 0.00 | 0.07 | 0.01 | 0.08 | 0.04 | 0.02 | *Planctomycetes* | *Planctomycetacia* | *Pirellulaceae* | *Pirellula* |
| 110 | 0.01 | 0.01 | 0.02 | 0.01 | 0.03 | 0.03 | *Epsilonbacteraeota* | *Campylobacteria* | *Arcobacteraceae* | *Arcobacter* |
| 111 | 0.07 | 0.01 | 0.01 | 0.02 | 0.01 | 0.06 | *Proteobacteria* | *Deltaproteobacteria* | *unclassified_Deltaproteobacteria* | *unclassified_Deltaproteobacteria* |
| 112 | 0.01 | 0.01 | 0.04 | 0.04 | 0.06 | 0.18 | *Bacteroidetes* | *Bacteroidia* | *37-13* | *37-13* |
| 113 | 0.04 | 0.00 | 0.00 | 0.01 | 0.00 | 0.00 | *Fibrobacteres* | *Fibrobacteria* | *Fibrobacteraceae* | *uncultured* |
| 114 | 0.00 | 0.01 | 0.00 | 0.01 | 0.00 | 0.01 | *Proteobacteria* | *Gammaproteobacteria* | *Chitinimonadaceae* | *Chitinivorax* |
| 115 | 0.50 | 0.09 | 0.02 | 0.27 | 0.03 | 0.48 | *Bacteroidetes* | *Bacteroidia* | *Saprospiraceae* | *Haliscomenobacter* |
| 116 | 0.02 | 0.00 | 0.02 | 0.02 | 0.01 | 0.01 | *Firmicutes* | *Clostridia* | *Ruminococcaceae* | *Subdoligranulum* |
| 117 | 0.01 | 0.00 | 0.01 | 0.01 | 0.01 | 0.00 | *Proteobacteria* | *Gammaproteobacteria* | *Burkholderiaceae* | *unclassified_Burkholderiaceae* |
| 118 | 0.01 | 0.00 | 0.02 | 0.02 | 0.02 | 0.00 | *Firmicutes* | *Erysipelotrichia* | *Erysipelotrichaceae* | *Erysipelotrichaceae_UCG-003* |
| 119 | 0.01 | 0.03 | 0.01 | 0.05 | 0.01 | 0.07 | *Acidobacteria* | *Acidobacteriia* | *Solibacteraceae_(Subgroup_3)* | *Bryobacter* |
| 120 | 0.00 | 0.00 | 0.01 | 0.00 | 0.00 | 0.01 | *Proteobacteria* | *Gammaproteobacteria* | *Burkholderiaceae* | *unclassified_Burkholderiaceae* |
| 121 | 0.02 | 0.08 | 0.01 | 1.15 | 1.49 | 0.12 | *Spirochaetes* | *Leptospirae* | *Leptospiraceae* | *Leptospira* |
| 122 | 0.00 | 0.01 | 0.01 | 0.02 | 0.07 | 0.03 | *Proteobacteria* | *Deltaproteobacteria* | *Desulfobacteraceae* | *Desulfobacter* |
| 123 | 0.03 | 0.01 | 0.01 | 0.01 | 0.11 | 0.02 | *Bacteroidetes* | *Bacteroidia* | *uncultured* | *uncultured* |
| 124 | 0.01 | 0.04 | 0.01 | 1.27 | 0.15 | 0.33 | *Bacteroidetes* | *Bacteroidia* | *37-13* | *37-13* |
| 125 | 0.01 | 0.01 | 0.01 | 0.10 | 0.00 | 0.07 | *Bacteroidetes* | *Bacteroidia* | *37-13* | *37-13* |
| 126 | 0.05 | 0.10 | 0.01 | 0.14 | 0.24 | 0.21 | *Bacteroidetes* | *Bacteroidia* | *37-13* | *37-13* |
| 127 | 0.01 | 0.03 | 0.00 | 0.02 | 0.02 | 0.13 | *Chloroflexi* | *Anaerolineae* | *SBR1031* | *SBR1031* |
| 128 | 0.00 | 0.01 | 0.00 | 0.00 | 0.00 | 0.00 | *Proteobacteria* | *Gammaproteobacteria* | *Burkholderiaceae* | *unclassified_Burkholderiaceae* |
| 129 | 0.00 | 0.00 | 0.00 | 0.00 | 0.00 | 0.00 | *Proteobacteria* | *Gammaproteobacteria* | *Burkholderiaceae* | *unclassified_Burkholderiaceae* |
| 130 | 0.00 | 0.00 | 0.01 | 0.00 | 0.00 | 0.02 | *Proteobacteria* | *Gammaproteobacteria* | *Burkholderiaceae* | *unclassified_Burkholderiaceae* |
| 131 | 0.01 | 0.00 | 0.00 | 0.09 | 0.09 | 0.11 | *Proteobacteria* | *Alphaproteobacteria* | *Rhizobiaceae* | *Ochrobactrum* |
| 132 | 0.01 | 0.02 | 0.02 | 0.02 | 0.02 | 0.04 | *Bacteroidetes* | *Bacteroidia* | *Flavobacteriaceae* | *Flavobacterium* |
| 133 | 0.09 | 0.15 | 0.00 | 0.08 | 0.04 | 0.03 | *Proteobacteria* | *Deltaproteobacteria* | *Phaselicystidaceae* | *Phaselicystis* |
| 134 | 0.06 | 0.02 | 0.00 | 0.02 | 0.02 | 0.02 | *Planctomycetes* | *Phycisphaerae* | *Phycisphaeraceae* | *SM1A02* |
| 135 | 0.00 | 0.08 | 0.00 | 0.06 | 0.00 | 0.07 | *Proteobacteria* | *Alphaproteobacteria* | *Rhodobacteraceae* | *Rhodobacter* |
| Total abundance | 15.92 | 14.56 | 19.88 | 24.05 | 24.77 | 26.20 |  |  |  |  |

**Table S6** Abbreviation and full name of bacteria in RDA diagram

| [*Abbreviation*](javascript:;) | [*Full*](javascript:;) [*name*](javascript:;) | [*Abbreviation*](javascript:;) | [*Full*](javascript:;) [*name*](javascript:;) |
| --- | --- | --- | --- |
| *Phen* | *Phenylobacterium* | *Lept* | *Leptospiraceae* |
| *Ferri* | *Ferribacterium* | *Vibr* | *Vibrionmonas* |
| *Lates* | *Latescibacteria* | *NS9* | *NS9_marine_group* |
| *Chit* | *Chitinivorax* | *BD1-7* | *BD1-7_clade* |
| *Novo* | *Novosphingobium* | *Phar* | *Phaeodactylibacter* |
| *Unclassified_R* | *Unclassified_Rhodocyclaceae* | *Elli* | *Ellin6067* |
| *Unclassified_S* | *Unclassified_Saprospiraceae* | *Aqua* | *Aquabacterium* |
| *Unclassified_B* | *Unclassified_Burkholderiaceae* | *Comp* | *Competibacter* |
| *Coma* | *Comamons* | *Chrys* | *Chryseolinea* |
| *Deni* | *Denitratisoma* | *Ther* | *Thermomonas* |
| *OM27* | *OM27_clade* | *Hali* | *Haliangium* |
| *Sub_10* | *Subgroup_10* | *Sub_6* | *Subgroup_6* |
| *Limn* | *Limnobacter* | *Flav* | *Flavobacterium* |
| *Accu* | *Accumulibacter* | *Tetr* | *Tetrasphaera* |
| *Rhod* | *Rhodoferax* | *Dech* | *Dechlormonas* |
| *Terr* | *Terrimonas* | *Sulf* | *Sulfuritalea* |
| *Nitroso* | *Nitrosomonas* | *Thau* | *Thauera* |
| *Ferru* | *Ferruginibacter* | *Nann* | *Nannocystis* |
| *Zoog* | *Zoogloea* | *Dokd* | *Dokdonella* |
| *Nitrosp* | *Nitrospira* | *Azoa* | *Azoarcus* |
| *AKYH* | *AKYH767* |  |  |

**Table S7** Spearman's correlation coefficient (ρ) at genus level

| Genus | Genus | ρ | Genus | Genus | ρ |
| --- | --- | --- | --- | --- | --- |
| *[Agitococcus]_lubricus_group* | *unclassified_Burkholderiaceae* | 0.943 | *Aquabacterium* | *AKYH767* | -0.829 |
|  | *Nitrosomonas* | 0.886 |  | *Haliangium* | -0.886 |
|  | *Dechloromonas* | 0.829 | *Azoarcus* | *OM27_clade* | -0.943 |
|  | *NS9_marine_group* | -0.886 | *Comamonas* | *Terrimonas* | -0.886 |
|  | *Thauera* | 0.943 | *Ferruginibacter* | *OM190* | -0.829 |
| *BD1-7_clade* | *Nitrosomonas* | 0.886 |  | *Subgroup_10* | -0.829 |
|  | *Dechloromonas* | 0.886 |  | *Leptothrix* | -0.829 |
|  | *Rhodoferax* | 0.812 |  | *Denitratisoma* | -0.886 |
|  | *Zoogloea* | 0.886 | *Flavobacterium* | *Thermomonas* | -0.886 |
|  | *[Agitococcus]_lubricus_group* | 0.886 | *Limnobacter* | *Nitrosomonas* | -0.886 |
|  | *NS9_marine_group* | -0.886 |  | *Sulfuritalea* | -0.943 |
| *Candidatus_Accumulibacter* | *AKYH767* | 0.886 |  | *NS9_marine_group* | 0.886 |
| *Chitinivorax* | *Nitrosomonas* | 0.886 |  | *unclassified_Rhodocyclaceae* | -0.943 |
|  | *Rhodoferax* | 0.812 |  | *OLB12* | -0.886 |
|  | *[Agitococcus]_lubricus_group* | 0.829 |  | *OLB8* | -0.886 |
|  | *NS9_marine_group* | -0.886 |  | *[Agitococcus]_lubricus_group* | -0.829 |
|  | *Subgroup_6* | -0.943 | *Nannocystis* | *Saccharimonadales* | -0.886 |
|  | *Subgroup_10* | -0.943 |  | *Subgroup_10* | -0.829 |
|  | *Denitratisoma* | -0.829 |  | *Chryseolinea* | -0.928 |
| *Chryseolinea* | *Thermomonas* | 0.899 | *Nitrosomonas* | *NS9_marine_group* | -1 |
|  | *Saccharimonadales* | 0.928 |  | *Sulfuritalea* | 0.943 |
| *Denitratisoma* | *OM190* | 0.829 |  | *OLB12* | 0.829 |
| *Denitratisoma* | *Saccharimonadales* | 0.943 | *Nitrosomonas* | *Subgroup_6* | -0.943 |
|  | *Subgroup_10* | 0.943 | *Novosphingobium* | *Subgroup_6* | 0.886 |
| *Ellin6067* | *Subgroup_6* | 0.886 |  | *Phenylobacterium* | 0.886 |
|  | *Phenylobacterium* | 0.886 |  | *Sulfuritalea* | -0.829 |
|  | *Novosphingobium* | 1 |  | *unclassified_Rhodocyclaceae* | -0.829 |
|  | *Sulfuritalea* | -0.829 |  | *Vibrionimonas* | -0.88 |
|  | *unclassified_Rhodocyclaceae* | -0.829 | *OLB12* | *Sulfuritalea* | 0.943 |
|  | *Vibrionimonas* | -0.88 |  | *NS9_marine_group* | -0.829 |
| *Latescibacteria* | *OM190* | 0.943 |  | *Saccharimonadales* | -0.943 |
|  | *Leptothrix* | 0.943 |  | *Chryseolinea* | -0.812 |
|  | *PB19* | 0.886 |  | *Denitratisoma* | -0.886 |
|  | *Leptospiraceae* | -0.829 | *PB19* | *OM190* | 0.943 |
| *Leptospiraceae* | *Ferruginibacter* | 0.943 |  | *Candidatus_Competibacter* | 0.829 |
|  | *unclassified_Saprospiraceae* | 0.829 |  | *Subgroup_10* | 0.829 |
|  | *OM190* | -0.943 |  | *unclassified_Burkholderiaceae* | -0.886 |
|  | *Saccharimonadales* | -0.829 |  | *Thauera* | -0.886 |
|  | *Subgroup_10* | -0.886 | *PLTA13* | *OM190* | 0.943 |
|  | *Leptothrix* | -0.943 |  | *Saccharimonadales* | 0.829 |
|  | *Denitratisoma* | -0.943 |  | *Leptothrix* | 0.943 |
|  | *PB19* | -0.886 |  | *PB19* | 0.886 |
| *Leptothrix* | *OM190* | 1 |  | *Latescibacteria* | 0.886 |
|  | *Denitratisoma* | 0.829 |  | *unclassified_Burkholderiaceae* | -0.829 |
|  | *PB19* | 0.943 |  | *Thauera* | -0.829 |
| *OLB8* | *NS9_marine_group* | -0.829 |  | *unclassified_Rhodocyclaceae* | -0.829 |
|  | *Saccharimonadales* | -0.943 |  | *unclassified_Saprospiraceae* | -0.829 |
| *OLB8* | *Chryseolinea* | -0.812 | *PLTA13* | *Vibrionimonas* | -0.88 |
|  | *Denitratisoma* | -0.886 |  | *Leptospiraceae* | -0.829 |
|  | *Nitrosomonas* | 0.829 | *Rhodoferax* | *Subgroup_6* | -0.812 |
|  | *Sulfuritalea* | 0.943 | *Sulfuritalea* | *NS9_marine_group* | -0.943 |
|  | *OLB12* | 1 |  | *Saccharimonadales* | -0.829 |
|  | *unclassified_Saprospiraceae* | 0.943 | *unclassified_Burkholderiaceae* | *Candidatus_Competibacter* | -0.886 |
| *Phaeodactylibacter* | *SC-I-84* | -0.943 | *unclassified_Saprospiraceae* | *Saccharimonadales* | -1 |
|  | *Ferruginibacter* | 0.829 |  | *Subgroup_10* | -0.886 |
| *Phenylobacterium* | *Sulfuritalea* | -0.829 |  | *Sulfuritalea* | 0.829 |
|  | *Subgroup_6* | 0.829 |  | *OLB12* | 0.943 |
| *Subgroup_10* | *Saccharimonadales* | 0.886 |  | *Nannocystis* | 0.886 |
| *Subgroup_6* | *NS9_marine_group* | 0.943 |  | *Chryseolinea* | -0.928 |
|  | *Sulfuritalea* | -0.886 |  | *Denitratisoma* | -0.943 |
|  | *Subgroup_10* | 0.829 | *Vibrionimonas* | *OM190* | -0.941 |
| *Tetrasphaera* | *Sulfuritalea* | 0.829 |  | *Candidatus_Competibacter* | -0.82 |
|  | *Phenylobacterium* | -0.829 |  | *Leptothrix* | -0.941 |
|  | *Aquabacterium* | -0.829 |  | *PB19* | -0.941 |
|  | *OM27_clade* | 0.886 |  | *Latescibacteria* | -0.82 |
|  | *Haliangium* | 0.943 |  | *Thauera* | 0.88 |
| *Thauera* | *Candidatus_Competibacter* | -0.886 |  | *unclassified_Rhodocyclaceae* | 0.88 |
|  | *unclassified_Burkholderiaceae* | 1 |  | *Leptospiraceae* | 0.88 |
| *unclassified_Rhodocyclaceae* | *unclassified_Burkholderiaceae* | 0.829 |  | *unclassified_Burkholderiaceae* | 0.88 |
|  | *Sulfuritalea* | 0.829 | *Zoogloea* | *Dechloromonas* | 1 |
|  | *Thauera* | 0.829 |  | *[Agitococcus]_lubricus_group* | 0.829 |
| *OM27_clade* | *Sulfuritalea* | 0.829 |  |  |  |
